# Host-Parasite Interaction of Atlantic salmon (*Salmo salar*) and the Ectoparasite *Neoparamoeba perurans* in Amoebic Gill Disease

**DOI:** 10.3389/fimmu.2021.672700

**Published:** 2021-05-31

**Authors:** Natasha A. Botwright, Amin R. Mohamed, Joel Slinger, Paula C. Lima, James W. Wynne

**Affiliations:** ^1^ Livestock and Aquaculture, CSIRO Agriculture and Food, St Lucia, QLD, Australia; ^2^ Livestock and Aquaculture, CSIRO Agriculture and Food, Woorim, QLD, Australia; ^3^ Livestock and Aquaculture, CSIRO Agriculture and Food, Hobart, TAS, Australia

**Keywords:** Atlantic salmon (*Salmo salar*), *Neoparamoeba perurans*, amoebic gill disease, host-parasite interaction, immunity, aquaculture, dual RNA-Seq

## Abstract

Marine farmed Atlantic salmon (*Salmo salar*) are susceptible to recurrent amoebic gill disease (AGD) caused by the ectoparasite *Neoparamoeba perurans* over the growout production cycle. The parasite elicits a highly localized response within the gill epithelium resulting in multifocal mucoid patches at the site of parasite attachment. This host-parasite response drives a complex immune reaction, which remains poorly understood. To generate a model for host-parasite interaction during pathogenesis of AGD in Atlantic salmon the local (gill) and systemic transcriptomic response in the host, and the parasite during AGD pathogenesis was explored. A dual RNA-seq approach together with differential gene expression and system-wide statistical analyses of gene and transcription factor networks was employed. A multi-tissue transcriptomic data set was generated from the gill (including both lesioned and non-lesioned tissue), head kidney and spleen tissues naïve and AGD-affected Atlantic salmon sourced from an *in vivo* AGD challenge trial. Differential gene expression of the salmon host indicates local and systemic upregulation of defense and immune responses. Two transcription factors, *znfOZF-like* and *znf70-like*, and their associated gene networks significantly altered with disease state. The majority of genes in these networks are candidates for mediators of the immune response, cellular proliferation and invasion. These include *Aurora kinase B-like*, *rho guanine nucleotide exchange factor 25-like* and *protein NDNF-like inhibited*. Analysis of the *N. perurans* transcriptome during AGD pathology compared to *in vitro* cultured *N. perurans* trophozoites, as a proxy for wild type trophozoites, identified multiple gene candidates for virulence and indicates a potential master regulatory gene system analogous to the two-component PhoP/Q system. Candidate genes identified are associated with invasion of host tissue, evasion of host defense mechanisms and formation of the mucoid lesion. We generated a novel model for host-parasite interaction during AGD pathogenesis through integration of host and parasite functional profiles. Collectively, this dual transcriptomic study provides novel molecular insights into the pathology of AGD and provides alternative theories for future research in a step towards improved management of AGD.

## Introduction

Amoebic gill disease (AGD) remains a serious parasitic infection of farmed salmonids globally (1) and has been estimated to increase the cost of production by 20% in Tasmania (2–4). The disease is caused by the marine protozoan parasite *Neoparamoeba perurans*, which, upon attachment to the mucosal surface of the gill, causes a highly localized host response. The site of amoeba attachment is characterized by epithelial desquamation and edema, epithelial hyperplasia, fusion of secondary lamellae, and interlamellar vesicle formation (5). A reduction in chloride cells is also associated with clinical AGD and is closely linked to epithelial hyperplasia (6). An infiltration of inflammatory immune cells, such as neutrophils and macrophages, can be observed within the central venous sinus adjacent to AGD lesions (5, 7). This cellular response is grossly characterized by raised multifocal lesions on the gill surface (2) leading to inappetence, respiratory stress, and often fatal inflammatory branchialitis.

Previous studies have examined the transcriptomic response of the gill and other immunological organs to AGD using various approaches. While these studies have revealed important insights into the AGD host response, the exact nature of the inflammatory response within AGD-affected tissue has not been fully resolved. Certain studies have identified upregulation of key inflammatory and immune related genes, including *TNFα*, *CD8*, *CD4*, *MHCI* and *MHCIIα* (8) within AGD-affected tissue, while other studies have shown either downregulation or no differential expression of these genes (9–11). A number of studies have shown a mRNA upregulation of the pro-inflammatory cytokine *interleukin-1 beta* (*IL-1β*) at the site of AGD infection in both rainbow trout (9) and Atlantic salmon (12). Furthermore, in Atlantic salmon the expression of *IL-1β* mRNA was localized to filament and lamellar epithelium pavement cells within gills (12), and the upregulation of *IL-1β* appears to largely be restricted to within AGD lesions (11). The highly localized nature of the host response to AGD has also been demonstrated through microarray based-transcriptome profiling where downregulation of the *p53 tumor suppressor protein*, and associated transcripts were localized to the AGD lesion (13). Downregulation of immune pathways within AGD lesions has also been observed for antigen processing pathways. Indeed, Young et al. (10) identified a coordinated downregulation of the *MHCI* antigen presentation pathway and *interferon-regulatory factor 1* within the lesioned area of the gill. Similarly, a downregulation of transcripts related to apoptosis were also identified within AGD-affected gill (14). More recently, the importance of T helper cells in the immune response to AGD has been described. Indeed, *interleukin (IL)-4*, a key cytokine involved in the Th2 pathways was significantly upregulated primarily within the interbranchial lymphoid tissue of AGD-affected gill (15) and within AGD lesions (16). In contrast, genes involved in the Th1 pathway, which are primarily responsible for activation of macrophages, were downregulated. The authors go on to postulate that induction of the Th2 response may be associated with an allergic reaction caused by the parasite (15).

While significant effort and progress has been made to understand the host response to AGD, considerably less attention has been paid to understanding the behavior of the parasite. It was not until 2012 when virulent *N. perurans* was isolated, cultured and shown to generate AGD in a laboratory challenge model (17) that significant effort has been invested into understanding the biology of the parasite (18–23). Perhaps the most striking feature of *N. perurans*, along with all other *Neoparamoeba* spp. is that they harbor a eukaryotic endosymbiont phylogenetically related to *Perkinsela* sp (24). Genome sequencing of the related species *Neoparamoeba pemaquidensis* and its *Perkinsela* endosymbiont has demonstrated mosaic biochemical pathways between the two genomes, suggesting an interdependence between host and endosymbiont (25). Furthermore, bacteria and viruses may contribute to the pathogenicity and virulence of *N. perurans* which has been reported to harbor the pathogenic bacteria, *Vibrio* within its microenvironment (26). Increased pathogenicity and virulence on passage through amoeba hosts has been reported previously for the amoeba species, *Acanthamoeba castellanii* and *Acanthamoeba astronyxis* and the bacteria *Legionella pneumophilia* (27, 28). Viruses may also contribute to virulence in *N. perurans*, as amoeba have also been reported to host several giant viruses, including adenoviruses and enteroviruses (28). Despite our increasing knowledge of *Neoparamoeba* biology, many knowledge gaps exist particularly regarding how the amoeba reacts and behaves when interacting with the host. In many respects, this is due to a lack of specific tools and reagents to investigate amoeba biology during infection. One recent advance was the development of an *in vitro* model of AGD based on a rainbow trout gill derived cell line which can be stimulated with *N. perurans* and the host-parasite response profiled (29). While this model successfully generates host responses similar to those observed *in vivo*, the response of the parasite is yet to be fully integrated.

Transcripts derived from *N. perurans* as a single-celled eukaryotic organism are polyadenylated like the host mRNA. This means that the gene expression profile of the amoeba during infection can be profiled in parallel to the host using mRNA-based transcriptome sequencing. This approach, commonly referred to as dual RNA-Seq, facilitates gene expression changes to be profiled simultaneously in both the parasite and host (30), and can provide valuable information concerning the expression of virulence factors and immune evasion pathways. With this in mind, the present study applied a dual RNA-Seq approach to characterize the molecular events that occur within an AGD lesion, both from the host and parasite perspective. Compared to *in vitro* cultured *N. perurans* trophozoites, wild-type AGD associated *N. perurans* upregulated multiple gene candidates for virulence factors and a master regulator. Genes associated with invasion of host tissue, evasion of host defense mechanisms and formation of the mucoid lesion were also upregulated. In parallel, localized immune responses were observed in AGD lesions in gill tissue and other immunologically important organs. Finally, drawing both host and parasite transcriptomic responses together we propose a model for host-parasite interaction for AGD in Atlantic salmon.

## Materials and Methods

### AGD Inoculation and Sample Collection

All animal procedures were approved by the CSIRO Queensland Animal Ethics Committee (project 2017-35, 2018‐09 and 2017-36) under the guidelines of the Australian Code for the Care and Use of Animals for Scientific Purposes (31). Seawater was sourced *via* offshore spear pumps, filtered (~40 µm), ozonated (100 g O^3^/h) and ultraviolet treated (80 mJ/cm^2^) before entering the laboratory. Tasmanian Atlantic salmon were originally imported from the River Philip, Canada in the 1960’s. The all-female Atlantic salmon fry for this project were transported from a commercial Tasmanian hatchery to the Bribie Island Research Centre, Woorim, Australia. Fish were reared in a single cohort in freshwater recirculating systems prior to adaptation to the marine environment. Smolting was achieved by increasing the photoperiod to a 24 h light regime. After 4 weeks, the photoperiod was changed to 12:12 h light/dark and the salinity gradually increased from 3 ppt to 35 ppt overnight. Atlantic salmon smolt (naïve to AGD) with a mean weight of 218 g were habituated in a 5000 L seawater (35 ppt) tank at 16°C, dissolved oxygen (96-100% saturation), pH 7.8, and flow rate of 45 L/min. Fish were fed daily to satiation on a commercial diet (Nutra, Skretting P/L, Cambridge, Australia).

At seven days a subset of 50 animals were transferred to a 1000 L tank to remain naïve to AGD. The other 507 fish in the cohort were subject to an AGD challenge trial (32, 33) by exposure to wild-type gill associated trophozoites of *N. perurans*. Wild-type (as opposed to laboratory cultured) *N. perurans* were obtained by the natural exposure of 40 marine adapted Atlantic salmon to wild-type *N. perurans* through co-habitation with AGD-affected fish maintained in an independent re-circulated marine biosecure experimental tank system dedicated to this purpose. Wild-type *N. perurans* were originally introduced into the system from AGD-affected gills collected from a commercial farm in Tasmania under animal ethics approval. The day before induction of AGD in experimental fish, 1 L of seawater was collected and subject to centrifugation in 50 mL falcon tubes at 15°C for 20 min at 4500 x g in an Eppendorf5804R (Eppendorf, Germany) to recover *N. perurans*. The seawater was poured off between subsequent centrifugation rounds until *N. perurans* were resuspended in a final volume of 10 mL of sterile seawater. The total amoeba in 1 L of seawater was enumerated by averaging repeated trophozoite counts (n = 10) on a hemocytometer (33). To induce AGD in experimental fish for this study, the water flow was stopped, and the water volume halved, before introducing a volume of water from the recirculated system containing a final concentration of 100 N*. perurans*/L for 2 h before restarting the water flow.

The average gill score for the AGD challenge population was assessed in 36 of the 507 AGD-affected fish using the ordinal ranking methodology on a scale of 0 to 5 of gross AGD pathology across all 16 gill surfaces outlined in Taylor et al. (34). At 21 days post-infection four animals at an average gill score of 3.3 (cohort average 3.1) were sampled from the AGD-affected cohort. AGD was confirmed by presumptive gill scoring and further confirmed by gill histopathology in fish from the same cohort as described by Wynne et al. (32). Gill biopsies were collected using a sterile single-use 2 mm biopsy punch (33), directly from the lesion and a second biopsy approximately 10 mm distal to the lesion from AGD-affected fish. Gill biopsies were also collected from four naïve fish to serve as an AGD-unaffected control. For all fish, primary immune tissues including head kidney and spleen were excised using sterile micro-scissors. A list of all samples collected in this study is provided in **Table 1**. Approximately 100 mg of tissue was collected and placed in 1.5 mL tubes containing 1 mL of RNAlater (Sigma-Aldrich) before storage at -80°C. For comparison with the lesion biopsy samples, *N. perurans* wild-type gill associated trophozoites were isolated from the gills of AGD-affected Atlantic salmon and cultured according to the method by English et al. (35) in a 1% malt yeast broth (MYB; 0.01% (w/v) malt extract (Oxoid) and 0.01% (w/v) yeast extract (Oxoid) in filtered, sterile seawater). The floating trophozoite form of *N. perurans* was isolated from *in vitro* cultures by centrifugation at 12 000 × g for 8 min at 16°C and the pellet stored at -80°C. The culture acts as a proxy for the wild type *N. perurans* trophozoites as insufficient *N. perurans* from the challenge are able to be sourced from the water column. We note that all *N. perurans* used in this study originated from the same source. However, by contrasting the lesion with the culture condition where there are no host signals, this improves the reliability of identification of *N. perurans* genes in the host-parasite interaction. The same method was employed by Mohamed et al. (36) in their dual RNA‐seq study of a coral (*Acropora tenuis*) and its symbiont (*Cladocopium goreaui*) during the establishment of symbiosis.

**Table 1:** Attributes of samples collected in this study.

Sample identifier	Tissue	Animal Condition	Description
C_GILL_F1	Gill	Naïve	Gill biological replicate 1
C_GILL_F2			Gill biological replicate 2
C_GILL_F3			Gill biological replicate 3
C_GILL_F4			Gill biological replicate 4
INF_D_GILL_F5		AGD-affected	Gill biopsy distal to the lesion biological replicate 1
INF_D_GILL_F6			Gill biopsy distal to the lesion biological replicate 2
INF_D_GILL_F7			Gill biopsy distal to the lesion biological replicate 3
INF_D_GILL_F8			Gill biopsy distal to the lesion biological replicate 4
INF_LES_F5	Gill & *Neoparamoeba perurans*		Gill biopsy at the lesion biological replicate 1
INF_LES_F6			Gill biopsy at the lesion biological replicate 2
INF_LES_F7*			Gill biopsy at the lesion biological replicate 3
INF_LES_F8			Gill biopsy at the lesion biological replicate 4
C_HK_F1	Head kidney	Naïve	Head kidney biological replicate 1
C_HK_F2			Head kidney biological replicate 2
C_HK_F3			Head kidney biological replicate 3
C_HK_F4			Head kidney biological replicate 4
INF_HK_F5		AGD-affected	Head kidney biological replicate 1
INF_HK_F6			Head kidney biological replicate 2
INF_HK_F7			Head kidney biological replicate 3
INF_HK_F8			Head kidney biological replicate 4
C_SP_F1	Spleen	Naïve	Spleen biological replicate 1
C_SP_F2			Spleen biological replicate 2
C_SP_F3			Spleen biological replicate 3
C_SP_F4			Spleen biological replicate 4
INF_SP_F5		AGD-affected	Spleen biological replicate 1
INF_SP_F6			Spleen biological replicate 2
INF_SP_F7			Spleen biological replicate 3
INF_SP_F8			Spleen biological replicate 4
FL_CELLS_1	*Neoparamoeba perurans*	Floating trophozoite	Cultured *N. perurans* floating trophozoites biological replicate 1
FL_CELLS_2			Cultured *N. perurans* floating trophozoites biological replicate 2
FL_CELLS_3			Cultured *N. perurans* floating trophozoites biological replicate 3

*Sample INF_LES_F7 failed RNA quality control and was excluded from the study.

C, control animal not affected by AGD; F#, fish number; INF, AGD-affected; D, biopsy distal to the lesion; LES, lesion biopsy; HK, head kidney; SP, spleen.

### Sample Preparation and Sequencing

**Figure 1:**
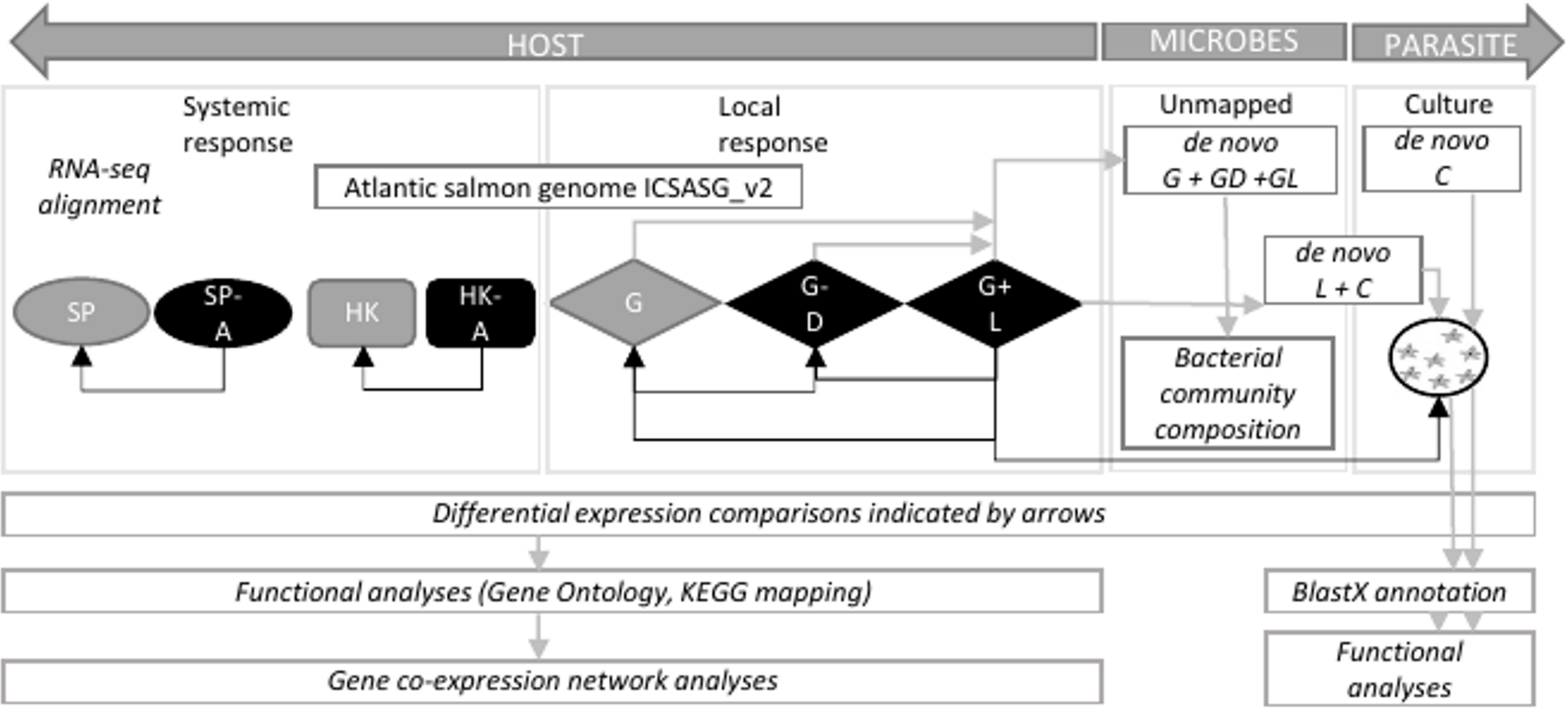
Schematic showing datasets and analytical approach to infer host-parasite interaction in amoebic gill disease (AGD). HOST, Atlantic salmon (*Salmo salar*); PARASITE, *Neoparamoeba perurans*; SP, spleen; A, AGD-affected; HK, head kidney; G, gill; D, distal to the lesion; L, lesion; C, cultured floating *N. perurans* trophozoites.

Total RNA was isolated using RNeasy mini kit (Qiagen). Tissues and the amoeba pellet were lysed in 450 µL of lysis solution on a Precellys 24 homogenizer (Thermo Fisher Scientific) for 2 x 30s at 4.0/ms. RNA was bound to a column and washed twice before elution with 40 µL RNase-free water at room temperature. RNA samples were treated with DNase to remove contaminating DNA using Turbo-DNase (Ambion). RNA quantity and quality were assessed using a NanoDrop ND-1000 spectrometer (Thermo Fisher Scientific) and Agilent 2100 bioanalyzer (Thermo Fisher Scientific). One of the gill lesion samples failed DNA quality analysis and was excluded from RNA-Seq library preparation. This resulted in preparation of a total of 30 RNA-Seq libraries from 11 gill biopsy samples (4 x naïve; 3 x lesion and 4 x distal to the lesion samples from 4 AGD-affected fish), eight head kidney, eight spleen (4 x naïve, 4 x AGD-affected fish) and three *in vitro* cultured floating *N. perurans* trophozoites samples. All libraries were prepared using the TruSeq RNA Sample Preparation Kit (Illumina). Host libraries were subject to 150 base pair, paired ended (PE) sequencing on the Illumina Novaseq 4000 sequencing platform at the Australian Genome Research Facility (AGRF) in Melbourne, Australia. *In vitro* cultured floating *N. perurans* trophozoites libraries were subject to 150 base pair PE sequencing on the Illumina HiSeq 4000 together with a second independent study (unpublished) undertaken concurrently with this project to conserve resources. Extensive data curation to remove microbial and host contamination from the *N. perurans* datasets reduces the impact of the different sequencing technologies as described in the section *Systemic Host Response*. A schematic of the datasets and analyses in this study is reported in **Figure 1**.

### Dual RNA-Seq Data Analysis

Illumina reads were checked for quality using FastQC version 0.11.8 (37) for all datasets. High quality reads (Q>30) were mapped to the Atlantic salmon genome ICSASG_v2 (38) using HISAT2 version 2.1.0 (39) with default parameters. Alignment files in BAM format were sorted by read name and converted into SAM format using SAMtools version 1.4 (40). Reads uniquely mapped to the salmon genome were extracted from BAM files based on MAPQ > 10 using SAMtools. The Python package HTSeq version 0.7.2 (41) was applied to count unique reads mapped to exons using the *reverse* parameter for strandedness. The gill lesion samples contained RNA from both the host and the parasite, therefore we used a stepwise *in silico* mapping approach to separate the reads belonging to each species. Reads were first mapped to the salmon genome to sort unmapped reads into three groups including 1) unmapped read whose mate is mapped; 2) mapped read whose mate is unmapped; and 3) both paired reads are unmapped. Reads were extracted using SAMtools and Bam2fastq options in BEDTools version 2.29.2 (42).

These unmapped reads were subject to a *de novo* transcriptome assembly with Trinity v 2.8.4 (43) to identify amoeba-related genes associated with the lesion. A second *de novo* transcriptome Trinity assembly was carried out with unmapped gill lesion and *in vitro* cultured *N. perurans* trophozoites reads to undertake differential analyses. Both assemblies followed the assembly method recommended by Hass et al. (43) for strand specific RNA-Seq libraries, minimum contig length of 500 base pairs and read normalization set to a depth of 30. Each *de novo* transcriptome assembly was further curated by annotating all contigs using Diamond version 0.9.31 (44) and blastx to search the NCBI non-redundant database (12/2019) for the top hit with an expected value (e-value) threshold of <1 x 10^-5^. Contigs were manually curated further based on the taxonomic classification of the annotated sequences using the NCBI taxonomy database (45). The broad categories for curation of each of these transcriptomes were defined as fish, amoeba/kinetoplastid endosymbionts and protozoans, bacteria and other, which were retained for further downstream analyses and interpretation of the host-parasite response. Raw reads were mapped to the *de novo* unmapped gill lesion transcriptome using RSEM version 1.3.0 (46) to validate transcripts due to the low quantity of amoeba data generated during sequencing.

The mRNA enrichment step prior to library preparation for sequencing should exclude bacterial genes as they are poorly polyadenylated, although this is not always the case (47). In this instance, the ‘contaminants’ are more likely to be endogenous in origin from the microbiome of the host, or *N. perurans*, or the culture media. Therefore, opportunistic assessment of the microbiome community present in the lesion samples is possible in the context of published data on AGD. Initially the bacterial species associated with gill lesion were identified in the *in vitro* cultured and gill lesion *de novo* transcriptome assembly as described above (**Figure 1**). Following the successful identification of species known to be associated with the gill microbiome of AGD-affected fish, a second *de novo* transcriptome assembly was generated using Trinity, applying the same parameters as the *N. perurans* transcriptome to contrast with naïve fish and the gill microbiome distal to the lesion of AGD-affected fish. Unlike the previous *N. perurans* transcriptome, this assembly included all unmapped raw reads from the gill including those generated from the naïve fish, biopsies distal to AGD lesions, and the AGD lesion data. While the *N. perurans* transcriptome had the advantage of *in vitro* cultured *N. perurans* to increase the depth of coverage for the amoeba, the unmapped (or bacterial microbiome only) transcriptome was limited in its input data to less than 10% of gill sequenced reads. However, the intent of this component of our study was to identify the major bacterial species that presented in conjunction with AGD at significant quantities to bypass the mRNA enrichment step prior to library enrichment, not the complete diversity of the microbiome which is more aptly assessed through microbial 16S sequencing. The *de novo* transcriptome assembly was curated for bacterial sequences by annotating all contigs using Diamond version 0.9.31 (44) and blastx to search the NCBI non-redundant database (12/2019) for the top hit with an expected value (e-value) threshold of < 1 x 10^-5^. Contigs were manually curated based on the NCBI taxonomic classification of the bacteria super kingdom. The relative proportions of each gene based on the transcript frequency (TPM) in the overall dataset were mapped for each bacterial candidate in the microbiome of the naïve and AGD-affected fish, including distal to AGD lesions and AGD lesion material.

### Differential Gene Expression, Clustering, and Functional Analyses

Differential gene expression among host tissues and curated *N. perurans* transcriptomes was inferred by analyzing the raw counts using edgeR version 3.20.9 (48) in R version 4.0.2 (49). To understand the local response to AGD pathology three comparisons for the gill biopsy samples were conducted. These were AGD lesion against naïve, distal to the AGD lesion against naïve, and lesion against distal to the AGD lesion. The systemic immune response was evaluated using the head kidney and spleen tissues, where comparisons were between AGD-affected samples and their respective naïve controls. For the parasite, the curated unmapped gill lesion, and the *in vitro* cultured *N. perurans* trophozoites transcriptomes were compared. P‐values were corrected using the Benjamini and Hochberg algorithm (50) for multiple testing. Differential gene expression (fold change > 2; P < 0.05) was compared between naïve and disease states. DEGs were hierarchically clustered using the normalized expression (CPM) values that were log_2_‐transformed and median‐centered by gene (43). Host data was further analyzed to infer function by gene ontology (GO) enrichment analyses. These were performed using the R package clusterProfiler version 3.9 (51) to identify enriched GO terms that belong to the three key GO categories [biological process (BP), cellular component (CC) or molecular functions (MF)] among the DEGs compared to a background set of expressed genes per tissue. GO terms with a corrected P‐value of < 0.05 were considered significant.

### Host Master Regulators, Gene Co-expression Networks, and Differential Connectivity Analyses

Key regulatory transcription factors (TF) contributing to differential expression in the AGD host response were assessed using regulatory impact factor (RIF) metrics (52). Data for putative TF genes in Atlantic salmon were obtained as described by Mohamed et al. (53). The normalized counts (CPM) of these TFs were retrieved for all host tissue samples from prior DEG analyses. Genes with a mean expression FPKM < 0.2 were excluded. These TFs were contrasted against the unique list of DEGs previously obtained for each tissue. The RIF approach comprises a set of two metrics designed to assign scores to regulatory genes consistently differentially co-expressed with target genes, and to those able to predict the abundance of target genes. Scores deviating ± 2.57 standard deviation (SD) from the mean were considered significant (corresponding to P < 0.01). Genes from differential expression and RIF analyses were selected based on overlap and mean normalized expression (at least 0.2 FPKM) to construct the networks. For gene network inference, genes were used as nodes and significant connections (edges) between them were identified using the Partial Correlation and Information Theory (PCIT) algorithm (54) to calculate the significance of the correlation between two nodes after accounting for all the other nodes in the network. An initial network was constructed using all samples and gene node connections (2SD; P < 0.05). To explore differential connectivity during AGD, two additional networks were constructed for naïve and AGD-affected samples. The number of connections per gene in relation to the naïve or AGD-affected network was computed enabling identification of differentially connected genes (DCGs), and subsequent review of subnetworks related to host-parasite interaction. Networks were visualized using Cytoscape Version 3.7.2 (55).

### Host-Parasite Interaction Pathway Analysis

To explore the host-parasite interaction further, gene pathways for the host and the parasite were independently visualized by submitting genes from differential expression analyses to the Kyoto Encyclopedia of Genes and Genomes (KEGG) pathway maps module (56–58). A specific parasitic response pathway from host data was not identified and led to further investigation of the KEGG database. This revealed that when selecting *Salmo salar* (sasa) as the organism potential human host-pathogen pathways are not automatically searched. Therefore, host and *N. perurans* genes were converted to KEGG pathway orthologues to enable exploration of the human amoebiasis pathway (hsa05146) associated with the amoeba parasite *Entamoeba histolytica* to infer a model for host-parasite interaction in AGD.

## Results

### Dual Transcriptome Sequencing and Assembly

Sequencing produced a total of 3.1 billion PE reads with approximately 81 million PE reads per Atlantic salmon library (**Supplementary Table 1**). The three lesion samples were sequenced to a higher depth producing approximately 362 M PE reads per library to recover transcriptomic data for *N. perurans* and the associated bacterial community in the presence of host tissue (**Supplementary Table 1**). Sequencing of the *N. perurans in vitro* cultured floating trophozoite samples produced approximately 35 M PE reads per library (**Supplementary Table 1**). An average mapping rate of 87% (70 M; gill lesion data 313 M) was achieved for all salmon samples to the Atlantic salmon reference genome ICSASG_v2 (38). The remaining 13% (47 M) of PE reads from lesion samples that did not map to the salmon genome were segregated for further analysis.

A *de novo* transcriptome assembly completed with only the gill lesion data produced 186,310 contigs with a re-mapping rate of 90%. NCBI taxonomy together with a blastx search against the non-redundant NCBI database was then used to classify the longest isoform for each gene (n = 56,657) into categories. A total of 35,306 contigs remained after classification, of which 77%, 2.4%, 0.6% and 20% of contigs contributed to host, *N. perurans* (and its endosymbiont), bacterial and ‘other’ species respectively. The ‘other’ category consisted of species matches that were considered to be taxonomically too diverse in origin, and of biological significance to provide insight into host-parasite interaction. This is a common issue encountered when working with non-model organisms. The low coverage of *N. perurans* and endosymbiont genes (n = 833) suggests that the 10% of *N. perurans* data recovered from the gill lesion was insufficient for a complete transcriptome to be assembled. Therefore, a second *de novo* transcriptome assembly was completed together with xenic *in vitro* cultured *N. perurans* trophozoites (106 M PE reads) and the unmapped gill lesion data (**Supplementary Table 1**). The initial assembly was 214,381 contigs before taxonomic classification annotated 144,214 contigs. Of these 84%, 6.5%, 1.5% and 8% of contigs contributed to host, *N. perurans* (and its endosymbiont), bacterial, and other species, respectively. The resultant amoeba and endosymbiont transcriptome contigs were extracted for downstream analyses.

### Local Host Response

**Figure 2:**
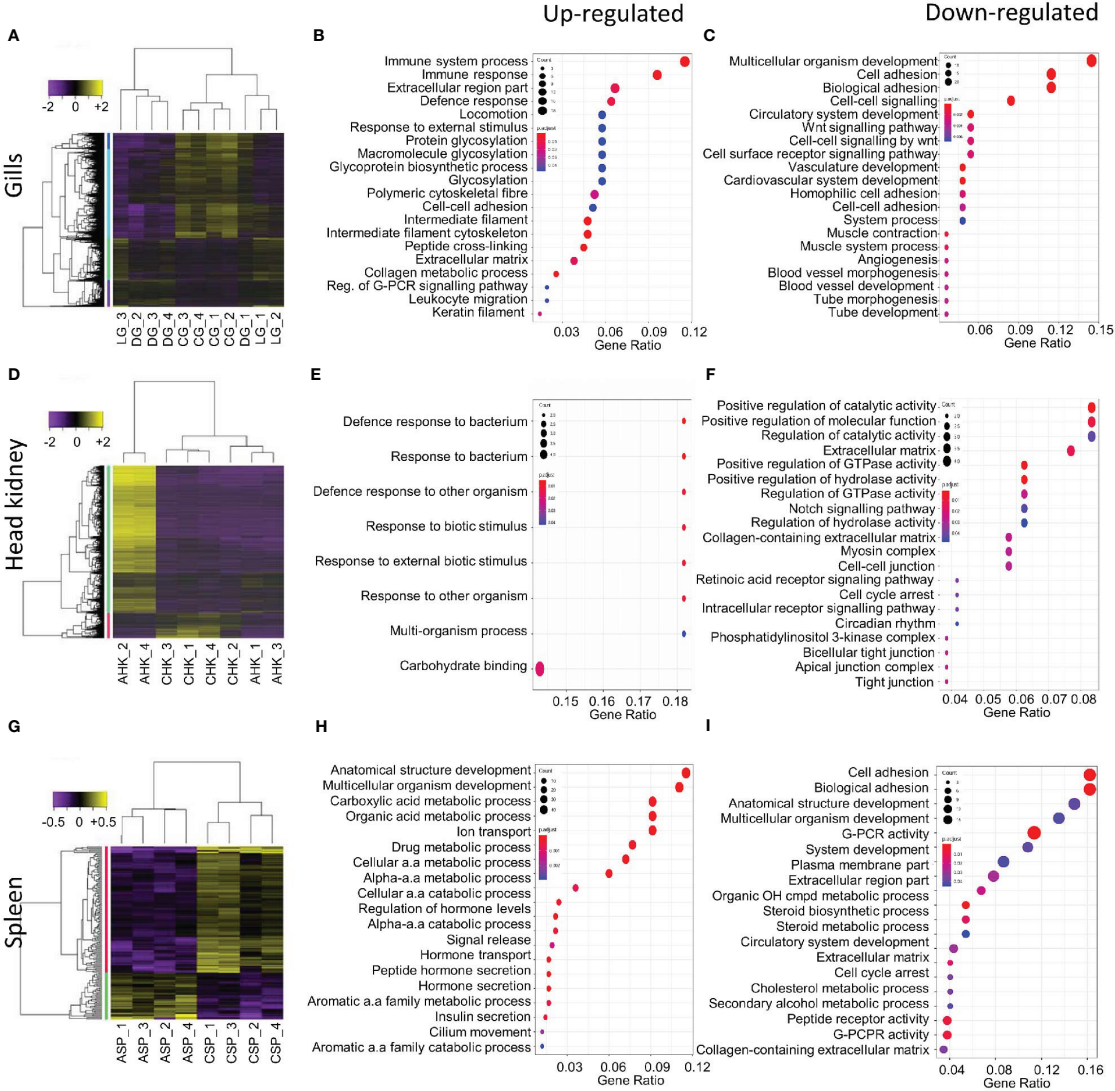
Summary of the host transcriptomic response during amoebic gill disease (AGD) progression. Differential gene expression and significantly enriched gene ontology (GO) terms are shown for the local **(A–C)** and systemic **(D–I)** response to AGD in Atlantic salmon. Heat maps show hierarchical clustering of differentially expressed genes (rows) with differential expression among replicates from a naïve (C) and AGD-affected (A) Atlantic salmon. Expression values are log_2_-transformed and median-centered by gene. **(A)** The local response is characterized by differential gene expression (fold change > 2; corrected P-value < 0.01) and includes a gill (G) biopsy distal (D) to the lesion (L) of an AGD-affected fish. The systemic response among head kidney (HK) **(D)** and spleen (SP) **(G)** replicates is characterized by differential gene expression (fold change > 2; corrected P-value < 0.05). Enriched gene ontology (GO) terms (hypergeometric test, Bonferroni-adjusted P < 0.05) among the differentially expressed genes for the gills **(B, C)**, head kidney **(E, F)** and spleen **(G, H)** along with the gene ratio for the genes that map to each term. The majority of the enriched terms are related to host defense and immune response.

Differential gene expression analysis was undertaken to compare the local host response in gill tissues of an AGD-affected fish compared to a naïve fish. This included the local response at the lesion and an area distal to the lesion. Hierarchical clustering of the biological replicates using Spearman correlations from a comparison of the normalized gene expression for all gill samples against each other showed that the biological replicates from the AGD lesion were most similar (**Supplementary Figure 1A**). In contrast, two of the biological replicates for the region distal to the lesion were similar to the lesion samples, while the third sample was similar to the naïve samples (**Supplementary Figure 1A**), suggesting the stage of disease progression may not have been as advanced for this fish. A total of 1,741 DEGs (fold change > 2; P < 0.01) were identified in the gill samples (**Figure 2A**). Of these, 644 DEGs were associated with the lesion compared to the control and 25 DEGs had a -log_(10)_FDR > 20 (**Supplementary Figure 1B**, **Supplementary Table 2**). In contrast, the region distal to the lesion exhibited 60 (**Supplementary Figure 1C**) and 49 DEGs (**Supplementary Figure 1D**) compared to the lesion and the naïve samples respectively (**Figure 2A**, **Supplementary Table 2**).

GO enrichment analysis was undertaken to identify the function of genes implicated in the local response to *N. perurans* invasion and subsequent AGD pathology. The top functional enrichment GO categories (BP, MF, CC) are shown in **Figures 2B, C** and **Supplementary Figure 3**. The significant GO terms and their corresponding genes are listed in **Supplementary Table 3**. In brief, enriched GO terms characterized by upregulated DEGs were consistent with activation and mobilization of innate immune system factors, inflammation, host defense and wound healing. While prospective pathogen invasion pathways were connected to the downregulation of genes associated with wnt and integrin-mediated signaling as well as cell adhesion (59–61). GO categories were heavily enriched for downregulated genes in developmental process categories indicative of diverting metabolic resources away from growth.

### Systemic Host Response

Differential gene expression analysis was undertaken in key immune organs in four biological replicates to assess the systemic host response of an AGD-affected fish compared to a naïve fish. For the head kidney, hierarchical clustering of the biological replicates based on normalized gene expression indicated that all naïve samples were closely correlated based on Spearman correlation. However, two of the AGD-affected head kidney samples clustered more closely with the naïve samples than their counterparts (**Supplementary Figure 2A**). In contrast, clustering of the spleen biological replicates clearly resolved AGD-affected from naïve samples (**Supplementary Figure 2B**). The transcriptomic response in the head kidney involved 1,463 DEGs (fold change >2; P < 0.05) (**Figure 2D**) of which 21 DEGs had a -log_(10)_FDR > 5 compared to the naïve samples (**Supplementary Figure 2C**; **Supplementary Table 4**). In the spleen, only 155 DEGs (fold change > 2; P < 0.05) were identified (**Figure 2G**) of which 20 had a -log_(10)_FDR > 5 compared to the naïve samples (**Supplementary Figure 2D**; **Supplementary Table 5**).

GO enrichment analysis was undertaken to identify the function of genes expressed in key immune organs (head kidney and spleen) implicated in the systemic response. The top functional enrichment GO categories (BP, MF, CC) are shown in **Figures 2E, F, H, I**. The significant GO terms and their corresponding genes are listed in **Supplementary Tables 6** and **7**. Enriched GO categories in the head kidney were similar to the local gill response with the downregulation of genes that modulate the inflammatory response, as well as signaling receptors and their pathways, that may assist pathogen invasion. A *mucin-13-like* gene associated with mucosal immunity was also identified in the head kidney (62). In contrast to the gill, GO term categories related to developmental processes were upregulated in the head kidney together with regulatory genes and transcription factors. In the spleen GO enrichment of upregulated genes corresponded to pathogen recognition with subsequent innate immune activation and acute inflammation. While GO enrichment for categories characterized by downregulated genes supported pathogen invasion, prevention of inflammation resolution, moderation of the innate immune response, and lesion suppression.

### Gene Co-expression Networks and Key Regulators of the Host Response to AGD

Transcription factors (TFs) are key regulators of gene expression in normal and disease states, however the detection and differential expression of TFs is often obscured by more highly expressed genes (52). Gene co-expression networks were used to characterize the transition between naïve and AGD disease states across all tissues of interest (63–67). Gene co-expression networks essentially seek the points of intersection between a single set of TFs and DEGs from all tissues in the study (e.g. gills, kidney, spleen). In this case, two gene networks are created, one for the naïve and a second for the AGD disease state. The transcription factors are not limited in the number of connections within or across tissues types. RIF metrics assign two scores to TFs (1) a score for TFs that are consistently differentially co-expressed with highly abundant and differentially expressed genes, and (2) TFs that have the ability to predict abundance of differentially expressed genes (52). This study contrasts differential gene expression data against predicted TFs to identify regulators of the local and systemic host response to AGD. A total of 403 TFs consisting of 174, 111 and 118 key TFs were identified in the gill, head kidney and spleen, respectively (**Supplementary Table 8**). Of these, 30 TFs were also differentially expressed in response to AGD (**Supplementary Table 9**). The top four included *Krueppel-like factor 15* in the head kidney (438 connections), f*ilamin-interacting protein FAM101A-like* (435 connections) and *peroxisome proliferator-activated receptor alpha* (336 connections) in the spleen and *early growth response protein 3* (261 connections) from the gill.

**Figure 3:**
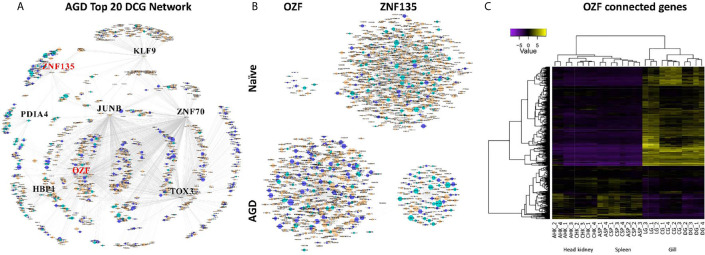
Sub-networks for the top differentially connected genes (DCGs) likely to regulate the transcriptomic response during amoebic gill disease (AGD) in Atlantic salmon. **(A)** AGD network of 8 transcription factors (TFs) among the top 20 DCGs using the PCIT algorithm. All nodes are represented by ellipses except for genes coding key regulators (TFs) which are diamond shaped. Nodes are orange for gill, green for head kidney and purple for spleen. The size of the nodes is relative to the normalized mean expression values in all samples. **(B)** Subnetworks of top differentially connected TFs. The networks created with the most differentially connected genes between naïve and AGD-affected networks with *zinc finger protein OZF-like* (*OZF*) as the key regulator with the greatest number of connections in the AGD-affected network, while *zinc finger 135* (*znf135*) lost the majority of its connections in the AGD-affected network. **(C)** Heat map shows hierarchical clustering of differential expression of connected genes (rows) in the OZF network among replicates from a naïve (C) and AGD-affected (A) Atlantic salmon in the head kidney (HK), spleen (SP) and gill (G). The AGD-affected gill data is represented by the lesion (L), a biopsy distal to the lesion (D) and naïve gill (C). Expression values (CPM) are log_2_-transformed and median-centered by gene.

The partial correlation and information theory (PCIT) algorithm is a statistical test used to identify significant correlations between genes and TFs (54). The PCIT algorithm was used to co-analyze key TFs and DEGs to infer gene networks in naïve and AGD disease states. Initially every gene was applied to each disease state to create a network comprising 1,864 genes with 883,408 connections. Visualization of the connections between the gene networks in Cytoscape enabled tissues types to be identified by color, and genes and TFs to be identified by shape and size depending on the number of connections. In the AGD disease state, the genes with the most connections (networks) were identified in the gill (n = 978; 53%) with networks from the head kidney (n = 406; 22%) and spleen (n=450; 25%) contributing equally to the overall host response to AGD. The top four genes based on the number of connections in the gene network in AGD-affected fish were from the gill (n = 3) and head kidney (n = 1) and included *aurora kinase B-like*, *zinc finger protein OZF-like*, *zinc finger protein 70-like* and *protein NDNF-like*, respectively. Key regulators undergo a substantial change in their number of connections during changes in disease state. Therefore, separate networks were constructed using 12 naïve samples and 15 AGD-affected samples, before identifying genes that underwent the largest change in connectivity (68) (**Figure 3**). The top 20 differentially connected genes (DCGs) and their putative roles are reported in **Table 2**. These include eight TFs, of which three belong to the zinc finger (znf) family and were more expressed in the gill (compared to other tissues) indicating a putative role in AGD progression. The most highly connected regulators based on increase (+) or decrease (–) in size of their connected gene networks were *znfOZF-like* (+537 connections) and *znf70-like* (+507 connections), while the least connected was *znf135-like* (-789 connections) in AGD.

The sub-network associated with *znfOZF-like* TF consisted of a total of 558 genes of which 386, 55 and 117 regulatory genes were identified in the gills, head kidney and spleen, respectively. This included a total of 142 TFs with 101 identified in the gill (**Supplementary Table 10**). Interestingly five (106573105, 106578124, 106586175, 100136550, 106608858) of the top 10 upregulated DCGs were in the *znfOZF-like* gene network (**Tables 2** and **3**). Further investigation of the 386 genes expressed in the gill identified 12 genes with potential roles in host-parasite interaction (**Table 3**). These included *hemagglutinin and amebocyte aggregation factor-like isoform X1* (90), *macrophage mannose receptor 1-like* (91), *T-cell immunoreceptor with Ig and ITIM domains*, the Ap-1 complex and TFs (*jun*, *fos*) (75, 95), *cytolysin RTX-A-like* (94), *mucin-2-like isoform X2* (93), *coxsackievirus and adenovirus receptor homolog* (92), *secreted frizzled-related protein 2-like* (85) and *T-lymphoma invasion and metastasis-inducing protein 2* (96).

**Table 2:** Top 20 differentially connected genes associated with amoebic gill disease (AGD) pathology in Atlantic salmon. Data for tissue of maximum expression, differential connectivity between naïve and AGD disease states, and mean expression are shown.

GeneID	Tissue	TF	ΔConnectivity	DE	Mean	Gene description	Putative role
*Increased connections in AGD-affected network*							
106573105	G	–	609	✓	3.164	*aurora kinase B-like*	Inhibit pathogen invasion (69)
106575349	G	✓	537	–	2.374	*zinc finger protein OZF-like*	DNA replication under stress
106560250	HK	–	524	✓	1.180	*protein NDNF-like*	Inhibit pathogen invasion (70)
106578124	G	✓	507	–	1.597	*zinc finger protein 70-like*	Inhibit invasion and proliferation (71)
106562393	S	✓	490	–	1.104	*TOX high mobility group box family member 3-like*	Activate CD8+ cytotoxic T lymphocytes; inhibit pathogen invasion (72, 73)
100194566	S	–	488	✓	4.863	*muscle specific ring finger protein 1-like*	proteasome-mediated degradation of pathogen proteins (74)
106564566	G	✓	484	–	5.658	*transcription factor jun-B-like*	AP-1 signaling in response to pathogen invasion (75)
106586175	S	–	483	✓	5.911	*catalase-like*	Tissue repair, oxidative stress response, host invasion (76)
100136550	G	–	482	✓	1.621	*insulin-like growth factor (IGF) 2b*	Tissue repair, IGF-mediated pathogen invasion (77)
106608858	S	–	470	✓	3.242	*svep1 sushi, von Willebrand factor type A, EGF and pentraxin domain containing 1*	Inhibit invasion and proliferation (78)
*Decreased connections in AGD-affected network*							
100194948	G	–	-878	✓	3.307	*CEF-10*	Oxidative stress (79)
106561981	G	–	-874	✓	3.161	*CKLF-like MARVEL transmembrane domain-containing protein 4*	Inflammatory response (80)
100136434	G	–	-799	✓	3.226	*matrix Gla protein*	Chronic inflammation (81, 82)
106587822	G	–	-797	✓	1.110	*ceramide synthase 2-like*	Inhibit invasion and replication (83)
106575389	G	✓	-789	–	2.819	*zinc finger protein 135-like*	Cytoskeleton organization (84)
106602777	G	–	-788	✓	1.105	*uncharacterized LOC106602777*	Uncharacterized
106573037	HK	✓	-785	–	3.052	*HMG box-containing protein 1-like*	P13K/Akt and wnt pathway; pathogen invasion (85, 86)
106602470	G	✓	-774	–	2.380	*Krueppel-like factor 9*	Inhibits IGF-mediated invasion of tissue (87)
106572685	G	–	-760	✓	3.391	*rho guanine nucleotide exchange factor 25-like*	Promote tissue invasion (88)
106578385	HK	✓	-754	–	5.824	protein disulfide isomerase family A, member 4	MHCI/antigenic peptides (89)

TF, transcription factor; DE, gene differential expression; G, gill; HK, head kidney; S, spleen. DE, gene differential expression.

**Table 3:** Selected genes expressed (fold change > 2; P < 0.05) in the gills from the z*inc finger OZF-like* gene regulatory network with potential activity in the host-parasite response to amoebic gill disease (AGD).

Description	Gene ID	DE	Mean	Putative role
*hemagglutinin/amebocyte aggregation factor-like isoform X1*	106576185	✓	1.364	Agglutination of host blood and aggregation of amoeba to form a lesion (90)
*macrophage mannose receptor 1-like, partial*	106561265	✓	2.123	Pathogen pattern recognition receptor (91)
*coxsackievirus and adenovirus receptor homolog*	106579874	✓	2.932	Viral receptor (92)
*mucin-2-like isoform X2*	106608496	✓	2.030	Host defense (93)
*cytolysin RTX-A-like*	106601042	✓	3.464	Host defense (94)
*T-cell immunoreceptor with Ig and ITIM domains isoform X2*	106575638	✓	1.567	Host defense (95)
*secreted frizzled-related protein 2-like*	106610025	✓	1.342	Wnt signaling pathway/pathogen invasion (85)
*T-lymphoma invasion and metastasis-inducing protein 2*	106607670	✓	2.595	Attachment and invasion (96)
*jun dimerization protein 2-like*	106611045	–	1.743	AP-1 signaling pathway in response to pathogens (75)
*fos-related antigen 2-like isoform X2*	106602906	✓	5.610	AP-1 signaling pathway in response to pathogens (75)
*AP-1 complex subunit mu-2-like*	106564498	✓	2.265	AP-1 signaling pathway in response to pathogens (75)

### *Neoparamoeba perurans* Gene Candidates for Host-Parasite Interaction

To infer candidate genes for host-parasite interaction an initial *de novo* assembly was generated based on the non-host gill lesion data to produce a total of 833 N*. perurans* and endosymbiont candidate genes after curation using blastx (e-value <1 x 10^-5^) to annotate the genes, and species classification to remove host and commensal bacteria genes. The majority of annotated genes from amoeba species matches were represented by *Acanthamoeba castellani*, *Dictyostelium discoideum*, *Neoparamoeba pemaquidensis*, *Naegleria gruberi* and *Tieghemostelium lacteum*, while the kinetoplastid endosymbiont was represented by *Perkinsela* sp. CCAP 1560/4. Pathogenic protozoans were also retained as candidates for virulence and pathogenicity as knowledge of these factors in AGD pathogenesis is highly sought. The low number of transcripts generated by the *de novo* assembly suggests a low number of raw reads specific to *N. perurans* were generated during sequencing and may have hindered the assembly of a larger number of transcripts. Assembling gill lesion sequence data together with the *in vitro* cultured *N. perurans* floating data increased the number of amoeba species and kinetoplastid transcripts to 9,399 available for differential gene expression. Hierarchical clustering of the biological replicates using Spearman correlation from a comparison of the normalized gene expression for all samples against each other showed that the biological replicates associated with each condition were most similar (**Supplementary Figure 4A**).

Differential expression of *N. perurans* in the gill lesion biopsy compared to the *in vitro* cultured floating trophozoites was undertaken in three biological replicates (**Supplementary Figure 4**). A total of 375 annotated transcripts were significantly expressed DEG’s (fold change > 2; P < 0.05) and their genes are listed in **Supplementary Table 11**. Of these DEGs, 28 were expressed by the endosymbiont including two immune suppression genes, *Yop effector YopM* and *cyclophilin*. Two stress response genes were also identified *chaperone protein DNAj* and *heat shock protein 70*. The remainder were related to cellular processes. A short list of candidate *N. perurans* genes and their putative roles in host-parasite interaction are provided in **Table 4**. In brief, candidates for tissue invasion, host immune evasion, pathogenicity, virulence factors and their regulatory systems were identified. The *de novo N. perurans* transcriptome assembly revealed a further five gene candidates for host-parasite interaction and pathogenicity (**Supplementary Table 12**). These were *extracellular Cu/Zn-superoxide dismutase* for defense against the host response and environment (110). *AprA*, from a master regulatory pathway for virulence that also suppresses host defense (111). Two virulence factors, *prokumamolisin activation domain containing protein* and *prohibitin* (112, 113) and lastly a candidate gene export of virulence proteins, *vacuolar sorting protein SNF7* (114).

**Table 4:** Selected genes differentially expressed (fold change > 2; P < 0.05) in *Neoparamoeba perurans* in the pathogenesis of amoebic gill disease in Atlantic salmon.

Description	Orthologue	Species	Mean	Putative role
*ankyrin repeat-containing protein*	XP_004350176.1	*Cavenderia fasciculata*	-11.628	Secreted to mimic and manipulate host responses (97)
	XP_004346535.1	*Acanthamoeba castellanii*	-10.811	
*cathepsin B*	XP_002677623.1	*Naegleria gruberi*	-13.796	Degrades host membranes (98, 99)
*protein serine/threonine kinase*	XP_004346574.1	*Acanthamoeba castellanii*	-11.765	Subvert host defense processes (100)
	XP_004338705.1	*Acanthamoeba castellanii*	-12.084	
	KYQ90593.1	*Tieghemostelium lacteum*	-10.724	
	KYQ96647.1	*Tieghemostelium lacteum*	-12.567	
*signal peptide peptidase*	XP_004353164.1	*Acanthamoeba castellanii*	-11.602	Virulence and host defense suppression (101)
*vacuolar proton ATPase*	XP_004356732.1	*Acanthamoeba castellanii*	-13.750	Acidification of environment (102, 103)
		
*Fbox domain containing protein*	XP_004340580.1	*Acanthamoeba castellanii*	-12.788	UPS toxin production and virulence (104)
*hybrid cluster protein*	CUE99497.1	*Bodo saltans*	13.020	Defense against host ROS production (105)
*PhoPQ-activated pathogenicity-related protein*	XP_004362351.1	*Cavenderia fasciculata*	-4.927	Master regulator of virulence (106)
*putative MADS-box transcription factor*	KYQ93049.1	*Tieghemostelium lacteum*	-12.545	Regulate secretion of virulence factors (107, 108)
*Ras guanine nucleotide exchange factor*	XP_643999.1	*Dictyostelium discoideum*	-11.062	Regulatory switch between virulent and avirulent forms (109)
*Ras subfamily protein*	XP_004333589.1	*Acanthamoeba castellanii*	-12.374	Regulatory switch between virulent and avirulent forms (109)
*RasGEF domain containing protein*	XP_004356500.1	*Acanthamoeba castellanii*	-12.409	Regulatory switch between virulent and avirulent forms (109)
	XP_004333796.1	*Acanthamoeba castellanii*	-11.045	
	XP_004335849.1	*Acanthamoeba castellanii*	-12.019	

UPS, Ubiquitin proteosome system.

### Microbial Community Associated With AGD

**Figure 4:**
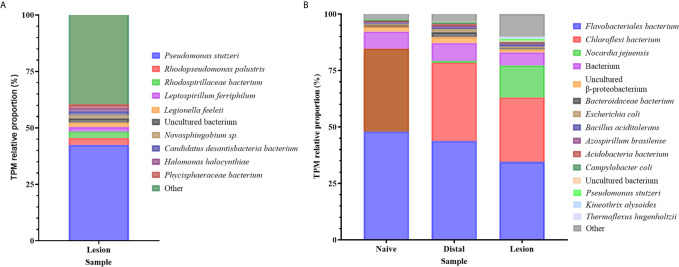
Bar plot of the bacterial taxa identified from sequence reads. **(A)** Bacterial taxa identified in the unmapped *Neoparamoeba perurans* transcriptome. While the community diversity of 142 species was plotted to show visual diversity, only the top 12 candidates based on proportional reads (>1% TPM) are denoted in the legend. **(B)** Bacterial taxa identified in the unmapped Atlantic salmon gill transcriptome from naïve fish, biopsies distal to AGD lesions, and the AGD lesion data. The top 15 candidates based on proportional reads (>1% TPM) are denoted in the legend.

Although the Illumina libraries used in this study were enriched for polyadenylated transcripts, a considerable number of bacterial reads were obtained and assembled, with functional annotations and taxonomic classifications from the NCBI database as described in the methods (**Supplementary Table 13**). An opportunistic assessment of bacterial species from these revealed a diverse abundance of many known marine-derived species in both the original *N. perurans* transcriptome and unmapped gill transcriptome. The relative proportions of each gene based on the transcript frequency (TPM) in the overall dataset were mapped for each bacterial candidate, overall demonstrating a high diversity of species. The most prevalent taxa based on standardized reads in this data are shown in **Figure 4**. Proportionally the taxa with higher read matrices in the original *N. perurans* transcriptome included *Pseudomonas stutzeri, Halomonas halocynthiae*, *Rhodopseudomonas palustris* and *Adhaeribacter aquaticus*, which are known marine bacterium (**Figure 4A**). The remaining top 10 most prevalent identifiable taxa have been identified in a number of aquatic environments, four are known to conform as a commensal species, while *Legionella feeleii* is a known pathogenic taxon. Interestingly, the *Epulopiscium* sp. has previously been identified as an intestinal endosymbiont taxon to surgeonfish *Naso tonganus* (115). The unmapped gill transcriptome data provided a similar story with the addition of naïve fish and AGD-affected data from gill biopsies, from distal to the lesion and the lesion (**Supplementary Table 13**). The majority of bacteria were detected in all sample types; however, the relative proportions were similar in the bacterial communities of the lesion and distal to the lesion transcripts of the AGD-affected fish compared to the naïve fish (**Figure 4B**). This is not surprising due to tank/environment effects that may have contributed to different microbial communities after 21 days in the flow through marine system. Known marine pathogenic bacteria were identified in the unmapped data set including *Nocardia jejuensis* and the Order *Flavobacteriales*, in addition to those previously mentioned. Interestingly, *Nocardia* was only identified in AGD-affected fish raising the question of whether it was present before, or after AGD appeared on the gill.

### Model for Host-Parasite Interaction

**Figure 5:**
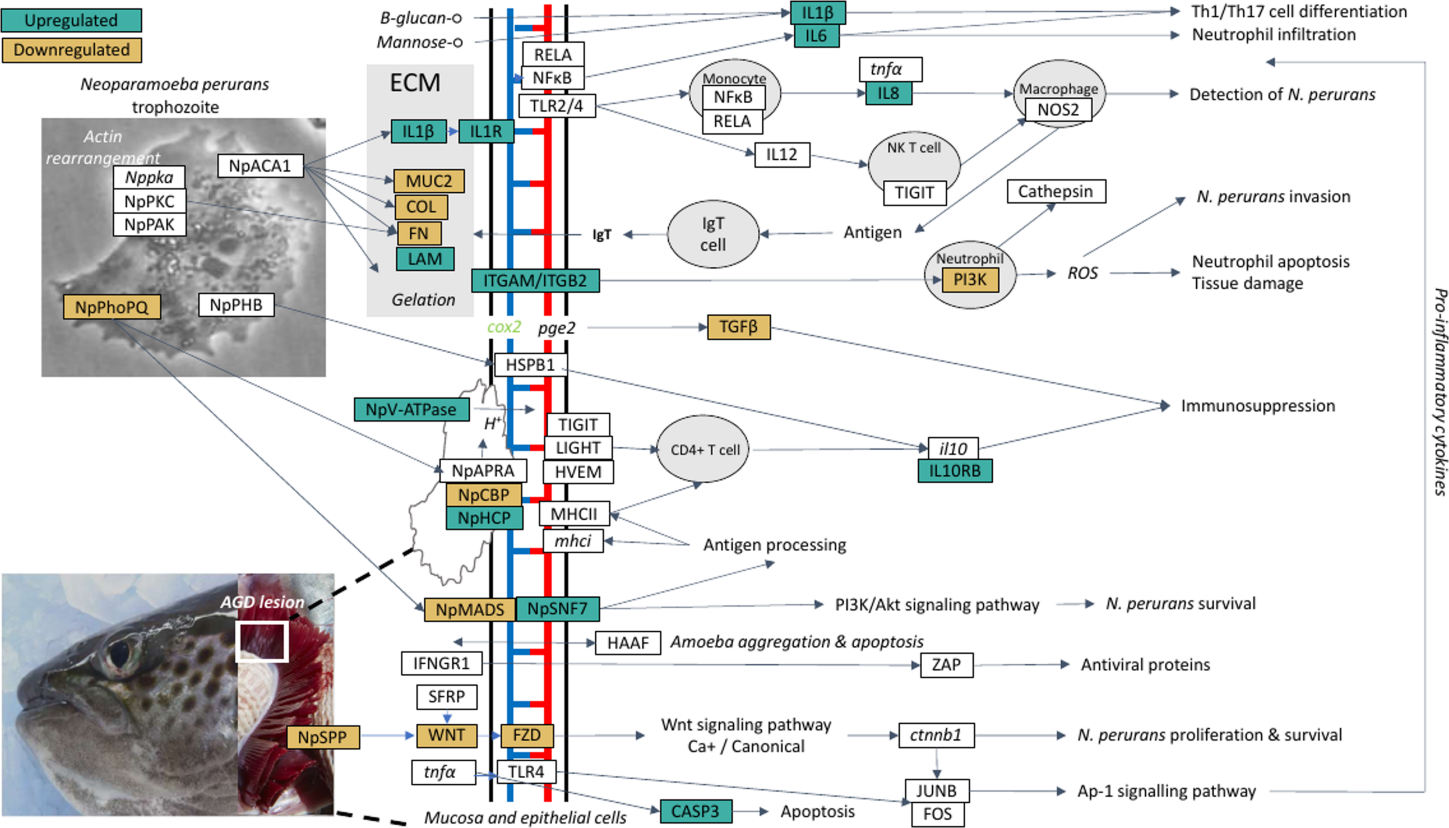
A theoretical model for the host-parasite interaction between *Neoparaomoeba perurans* candidate genes and Atlantic salmon during the pathogenesis of amoebic gill disease (AGD). The pathway was generated from the differential gene expression and network analyses together with the key KEGG pathways mapped and visualized (sasa04310, sasa05168, sasa05132, hsa04151, hsa05146) for the host and the parasite. Pathogen invasion is facilitated by degradation of host mucus and epithelia. To maintain a pathogen friendly environment on the gill *N. perurans* releases factors to decrease ammonia and reactive oxygen species released by the host. Actin rearrangement of the pathogen and the host facilitates attachment. Virulence factors are modulated by the pathogen master two component PhoPQ virulence regulatory system. Downregulation of the host wnt, Ap-1 and PI3K/Akt signaling pathways supports pathogen immune evasion, proliferation, and survival. The Th1/17 cell differentiation pathway is upregulated inducing innate and adaptive immune responses in the host. NP signifies *N. perurans* genes, upregulated genes are green, downregulated brown, not differentially expressed are white, italicized genes were not identified in our dataset. CASP3, *caspase-3*; COL, collagen (various); *cox2*, *cyclooxygenase 2*; *ctnnb1*, *β-catenin*; FN, *fibronectin*; FOS, *fos*; FZD, *frizzled*; HAAF, *hemagglutinin/amebocyte aggregation factor-like*; HSPB1, *heat shock protein beta-1-like*; HVEM, *tumor necrosis factor receptor superfamily*; IFNGR1, *interferon gamma receptor 1*; *il10*, *interleukin 10*; IL10RB, *interleukin-10 receptor subunit beta-like*; IL12, *interleukin-12*; IL1R1, *interleukin-1 receptor type 1-like*; IL1β, *interleukin-1 beta*; IL6, *interleukin-6*; IL8, *interleukin 8*; ITGAM, *integrin alpha-X-like*; ITGB2, *integrin beta-2*; JUNB, *junb*; LAM, *laminin*; LIGHT, *tumor necrosis factor ligand superfamily*; *mhci*, *major histocompatibility complex class I*; MHCII, *major histocompatibility complex class II*; MUC2, *mucin-2-like*; NFκB1, nuclear factor NF-κ-β p105 subunit-like; NOS2, nitric oxide synthase 2; *Np*ACA1, *prokumamolisin activation domain containing protein*; *Np*APRA, *AprA protease*; *Np*CBP, *cathepsin-B*; *Np*HCP, *hybrid cluster protein*; *Np*MADS, *MADS-box transcription factor*; *Np*PHB, *prohibitin*; *Np*PhoPQ, *PhoPQ-activated pathogenicity-related protein*; *Np*SNF7, *vacuolar sorting protein SNF7*; *Np*SPP, *signal peptide peptidase*; *Np*V-ATPase, *vacuolar proton ATPase*; *Np*PAK, *p-21 activated kinase*; *Nppka*, *protein kinase A*; *Np*PKC, *protein kinase C*; *pge2*, *prostaglandin E2*; PI3K, *phosphatidylinositol 3’-kinase*; RELA, *putative transcription factor p65 homolog*; SFRP, *secreted* frizzled-related protein; TGFβ, *transforming growth factor beta*; TIGIT, *T-cell immunoreceptor with Ig and ITIM domains*; TLR2/4, *toll-like receptor 2 and 4*; *tnfa*, *tumor necrosis factor alpha*; WNT, *protein Wnt*; ZAP, *zinc finger antiviral protein.*

A theoretical model for the host-parasite interaction in Atlantic salmon in response to AGD was inferred based on the data generated in this study and visualization in the KEGG pathways module for salmon, *Salmon salar* (sasa). The model generated from our data is shown in **Figure 5** and a list of the genes and their isoforms in the model is provided in **Supplementary Table 14**. In the local host response to AGD, visualization of KEGG pathways revealed downregulated genes involved in antigen processing and presentation through MHCI/MHCII complexes, the phosphatidylinositol 3’-kinase (PI3K)-Akt signaling pathway and the NF-κβ signaling pathway. In contrast, upregulated genes mapped to the C-type lectin and Toll-like receptor pathways, and the wnt and NOD signaling pathways. KEGG pathway mapping of the systemic response in the head kidney and spleen revealed that the majority of upregulated genes (3,151 and 335, respectively) were involved in pathways for metabolism, focal adhesion, C-type lectins, calcium and toll-like receptor signaling and cellular integrity. However, to a lesser extent wnt signaling, bacterial and viral infection, and immune pathways were also well represented. Downregulated genes in the head kidney and spleen (597 and 221, respectively) mapped predominately to metabolism pathways, followed by a multitude of signaling pathways including calcium, foxO, insulin, MAPK, apelin, Erb8 and p53. Interestingly, the cytokine-cytokine receptor interaction pathway was equally represented by upregulated and downregulated genes in the spleen.

Visualization and mapping of the top 20 DCGs (**Table 2**) against the KEGG pathways revealed that the top two gill TFs (*znfOZF-like*, *znf70-like*) were antiviral proteins in the Herpes simplex virus 1 infection pathway (sasa05168). Further exploration of the *znfOZF-like* gene network (**Figure 3**) revealed 26 genes in the Herpes simplex virus 1 infection pathway suggesting a viral-like response may be initiated in response to parasite invasion in AGD. Lastly, a review of genes mapping to components of bacterial and parasite pathways including the Ap-1 and PI3K-Akt signaling pathway (hsa04151) in Salmonella infection (sasa05132), and the human *Entamoeba histolytica* amoebiasis pathway (hsa05146), provided insight to the proposed host-parasite model for AGD. The model identifies a theoretical pathway for the regulation of virulence in *N. perurans*, plausible methods for modulating the host environment to support parasite proliferation and evasion of the host immune response.

## Discussion

In this study, a dual RNA-Seq approach together with differential gene expression, and system-wide analyses of TF networks was employed to present a theoretical model for the host-parasite interaction during pathogenesis of AGD in Atlantic salmon. A recent review by Marcos-Lopez and Rodger (116) discusses the host response to AGD in detail, therefore we will focus on key findings from our study that provide new insights into the host-parasite interaction, with a particular focus on the factors responsible for virulence, and evasion of the host immune response.

### Candidate Pathways for Parasite Propagation and Attachment

Differential gene expression, gene networks and regulatory transcription factors in this study suggest a possible mechanism for *N. perurans* to evade detection and facilitate propagation on attachment to the gill of AGD-affected Atlantic salmon. The BP GO term ‘Wnt signaling pathway’ was enriched in the gill and the head kidney with downregulated genes including protein *wnt-4a-*, *5b-* and *10a-like*, and *secreted frizzled related proteins 1-*, *2-* and *5-like*. There are three wnt signaling pathways including the canonical or β-catenin-dependent pathway, and the non-canonical pathways, planar cell polarity and wnt/Ca^2+^ (calcium) each of which interact with a frizzled transmembrane receptor to promote paracrine or autocrine signaling (117, 118). This pathway is involved in cell cycle control, cytoskeleton reorganization during phagocytosis and cell migration, autophagy, apoptosis, and inflammation (85). The canonical and wnt/Ca^2+^ signaling pathways were enriched in all host differential expression and network analyses in this study. Both intracellular and extracellular bacteria have evolved to modulate and evade the host innate immune response through subversion of these wnt pathways. This may occur through activation or inhibition of the wnt pathway that results in immune suppression, induction of inflammation, disruption to epithelia and promotion or suppression of cellular proliferation depending on the pathogens intra- or extra-cellular location to the host (85).

In our study, the PhoP/Q system was identified as a candidate for the master regulator of virulence factors in *N. perurans* through blast similarity with the amoebozoa cellular slime mold, *Cavenderia fasciculata* (*syn. Dictyostelium fasciculatum).* This system has been reported to induce pathogenic factors implicated in *Salmonella enterica* invasion of intestinal epithelium through suppression of the wnt-signaling canonical pathway (119). *N. perurans* exhibits downregulation of PhoP/Q factors in the lesion, however this may be due to the late stage of the AGD lesion in our study of gill index 3.4. In contrast to the canonical pathway that is activated through multiple wnt proteins, the wnt/Ca^2+^ pathway is activated only through the binding of wnt5 to frizzled (sasa04310). We propose that the candidate genes for *N. perurans* virulence factors which are secreted effector proteins, *secreted frizzled protein* (*NpSFRP*) and the *signal peptide peptidase* (*NpSPP*), may bind to wnt5 subverting the pathway to promote proliferation and survival of *N. perurans* during invasion. The wnt signaling pathway also interacts with the Ap-1 signaling pathway to induce innate and adaptive immune responses. Likewise, viruses reported to hijack this pathway induce overexpression of the *c-Jun* and *c-Fos* proteins. These proteins make up the dimeric Ap-1 TF whose dysregulation promotes carcinogenesis and tumor progression in cancer (75). A multitude of genes identified in this study have been reported to be involved in carcinogenesis or tumorigenesis in cancer, suggesting that dysregulation and overexpression of genes is a common point of intersection with AGD in Atlantic salmon.

### Gene Candidates for Host Pathogen Recognition and Sequestration in AGD

Investigation of the genes expressed in the gill identified several candidates for roles in host-parasite interaction (**Table 3**). Expression of *hemagglutinin and amebocyte aggregation factor-like isoform X1* is a candidate gene for sequestration and aggregation of *N. perurans* into a lesion and agglutination of host blood (90). Pathogens released by the parasite may be recognized by the lectin-C pattern recognition receptor, *macrophage mannose receptor 1-like* which also stimulates *interleukin-1β* and the Th1/Th17 pro-inflammatory cytokine response (91). Interestingly, the Th1, Th2 and Th17 cell differentiation pathways reported to be associated with AGD through quantitative trait locus (QTL) and quantitative PCR analyses, were not markedly enriched pathways in our analysis (15, 120). Furthermore, the data were contradictory with previous studies, exhibiting upregulation of Th17 genes and downregulation of several other genes in the Th1, Th2 and Th17 cell differentiation pathways leading to their expression. The genes of the Th1/Th17 pathway identified however suggest that the pathogen may be recognized by a lectin-C pattern recognition receptor, *macrophage mannose receptor 1-like* or *β-glucan receptor* which stimulates the Th1/Th17 pro-inflammatory cytokine response (91). The contradictory Th1/Th17 data provides support for using system wide analyses to unravel the mechanisms behind complex disease states. In future studies, the concurrent use of multiple datasets (tissues, timepoints, backgrounds, pathogen strains) and types (transcriptome, methylation) in system wide analyses may prove to be invaluable in progressing our understanding of AGD.

### Gene Candidates and Gene Networks in Host Defense Against AGD

The fish in this study had not been exposed to *N. perurans*, or commensal bacteria or viruses associated with AGD prior to participating in this study. Therefore, the adaptive immune system was not primed to recognize and respond to the specific pathogen/s associated with AGD, in the same manner that the immune system may respond on its second interaction with the disease following successful treatment. Valdenegro-Vega et al. (121) have previously reported that consecutive challenges with *N. perurans* resulted in elevated IgM gene expression at gill lesions occurring 31 days after infection. While the primary infection does not induce an IgM or IgT transcriptome response. This is suggestive of an inefficient adaptive immune response on first introduction to the *N. perurans* unrecognized pathogen. With this consideration, the transcriptome differential expression and regulatory transcription factor networks in our study indicate that the host induces the primary defense pathways for bacteria and virus defense at the first interaction with *N. perurans* (and any viruses or bacteria it may harbor) in AGD in naïve Atlantic salmon.

Gene network analysis is a system wide approach that is able to connect differential gene expression data from all tissues with their regulatory TFs. The gene co-expression network analyses in this study indicate that the TF *znfOZF-like* regulatory network is the most active in AGD, with five of the top 10 (106573105, 106578124, 106586175, 100136550, 106608858) upregulated DCGs contributing to this gene network (**Tables 2** and **3**). The znfOZF protein is a Kruppel type of nuclear zinc finger protein whose dysregulation has been implicated in tumor genesis in cancer (71, 122) suggesting its role in AGD is to prevent invasion and proliferation of *N. perurans*. We identified 26 genes in the Herpes simplex virus 1 pathway from the *znfOZF-like* regulatory network with a total of 18 genes coding for the anti-viral proteins (ZAP). Furthermore, all of the key regulatory TFs identified in the top 20 DCGs were also ZAP proteins. Each of the ZAP proteins is a *zinc finger transcription factor* (**Supplementary Table 14**). The Herpes simplex virus 1 pathway was also consistently identified in our local and systemic KEGG visualizations for the host differential expression data, suggesting a viral-like response may be initiated in response to parasite invasion in AGD. In contrast bacterial defense mechanisms are indicated by genes mapping to the KEGG Ap-1 and PI3K-Akt signaling pathways (hsa04151) in Salmonella infection (sasa05132). These pathways are also closely connected to the wnt signaling pathway, which we propose is a candidate host pathway for manipulation by *N. perurans* during AGD pathogenesis in our model for host-parasite interaction.

Other host defense mechanisms identified in the *znfOZF-like* regulatory network are *cytolysin RTX-A-like* and *mucin-2-like isoform X2*, which may protect the host cells against bacterial pore-forming toxins and production of an insoluble gel mucus barrier to protect cells against invasion, respectively (93, 94). Of the remaining TFs identified through the network analysis, one was upregulated in the spleen and two were downregulated in the head kidney. *TOX high mobility group box family member 3-like* (*TOX3*) in the spleen has been shown to inhibit the proliferation and migration of cancer cells by transcriptional regulation of *SNAI1* and *SNAI2* to prevent disruption of the epithelial cell layer (72). However, in the spleen *TOX3* is a regulator of innate lymphoid cells in particular the pathogen primed CD8+ cytotoxic T lymphocytes suggesting its role in AGD is activation of the immune system (73). In contrast, the *HMG box-containing protein 1-like* is a regulator of key pathways including the PI3K/Akt and wnt pathways which are downregulated in cancer and hijacked by parasites in the host-parasite interaction during invasion as previously discussed (85, 86). The final key regulatory TF identified in the head kidney is *protein disulfide isomerase family A, member 4* (*PDIA4*). According to KEGG pathway visualization *PDIA4* is responsible for the loading of antigenic peptides into MHCI molecules in the endoplasmic reticulum for release at the site of the infection (Sasa04141).

The majority of DCGs associated with AGD exhibited similar activities to those induced by the TFs, acting as mediators of the immune response, or roles in cellular proliferation and invasion. Eight of the 12 gene networks were more expressed in gill tissue with two upregulated and three downregulated in response to AGD. Three have been implicated in invasion of tissue in cancer including *Aurora kinase B-like*, *rho guanine nucleotide exchange factor 25-like*, *protein NDNF-like inhibited*, however, their differential expression suggests a preventative role in this instance (69, 70, 88). Two other genes, *svep1 sushi, von Willebrand factor type A EGF and pentraxin domain containing 1*, and *ceramide synthase 2-like* are reported to suppress tumors (78, 83, 123). While *muscle specific ring finger protein 1-like*, an E3 ubiquitin-protein ligase has been reported to be involved in proteasome-mediated degradation (74) which may be involved in degrading proteins originating from *N. perurans* in host defense. The inflammatory response to AGD is potentially dampened by the downregulated inflammatory mediated gene networks connected to the gene *CKLF-like MARVEL transmembrane domain-containing protein 4* which exhibits similar expression in lung cancer, and *matrix Gla protein* which has been implicated in chronic inflammatory diseases as well as lung cancer (80–82). Two of the identified regulatory gene networks are associated with tissue repair including *insulin-like growth factor 2b* and *catalase-like* (77). The latter is involved in the oxidative stress response and also regulates hydrogen peroxide metabolism (76). Interestingly, *Trypanosoma cruzi* has been reported to modulate the oxidative stress (FoxO signaling pathway) response to aid invasion of the host (79). Sustained production of reactive oxygen species (ROS) due to the *T. cruzi* infection coupled with an insufficient antioxidant response leads to long-term oxidative stress in the host. Furthermore, the integrin signaling-associated gene, *T. cruzi cyr61*, which is a homologue of *cef10* in *S. salar*, is downregulated in the late stages of *T. cruzi* infection after infiltration of host tissue (79).

### Gene Candidates in the Systemic Host Response to Late Stage AGD

The head kidney and spleen, while both lymphoid organs, have different roles in immunity (124). The approximate 9-fold increase in DEGs in the head kidney and spleen is consistent with the head kidneys dual roles in detoxification and the immune response (125). The head kidney responds indirectly to the external parasite through alteration of osmoregulation and excretion at the gill (125) in response to compromised gill physiology from the AGD lesion (5). It also has a direct immune response through the differentiation of leucocytes for general immunity (124). This reduced number of DEGs in the spleen compared to the head kidney reflects the spleens primary role to filter and maintain the red blood cell population in circulation (125), as there is not profuse bleeding at lesions on the gill.

The local host defense at the gill is supported by upregulation of genes coding for *cathelicidin antimicrobial peptides* and *lysozyme C II* in the spleen. These gene candidates have previously been identified in Atlantic salmon in response to AGD (14, 126, 127), as well as to the bacterial infection *Yersinia ruckerii* and the sea louse, *Lepeophtheirus salmonis* (14, 128, 129). In Atlantic salmon the C-type lectins have been reported to be upregulated during the first five days post infection in AGD (13, 29). While more recently, a glycan and lectin microarray study identified mannobiose and *N*-acetylgalactosamine as candidates for gill epithelium binding of *N. perurans* (130). Recombinant mannose-binding proteins have previously been reported to bind *N. perurans* and produce antibodies in the host (131). While *N*-acetylgalactosamine has also been reported to be involved in mucosal adherence for the pathogenic amoeba, *Entamoeba histolytica* (132). Gene candidates for the downregulation of immune BPs with GTPase activity, include the *ras-GEF domain-containing family member 1B-B-like* and *T-cell lymphoma invasion and metastasis 2* which are induced in macrophages in response to Toll-like receptor agonists (133) and participate in proliferation and invasion of tumors (96), respectively. In the spleen the MAPK pathway molecular functions were downregulated suggesting the potential for prevention of resolution of inflammation and lesion suppression in response to *N. perurans* invasion (134).

The systemic innate immune response in the head kidney was down-regulated through the *sphingosine 1-phosphate receptor 4-like receptor* (135) and *GPCR C3a anaphylatoxin chemotactic receptor-like* which causes migration of eosinophils, mast cells and macrophages to the site of injury as part of the complement cascade (136). Several genes coding for GPCRs were downregulated in the systemic response in both the head kidney and spleen including probable *CD97 antigen-like GPCR*, *G-protein coupled receptor 132*, *2-oxoglutarate receptor 1-like*, *G-protein coupled receptor 124-like* (wnt pathway), *chemokine-like receptor 1*, *C-C chemokine receptor type 9* and *C-X-C chemokine receptor type 4*. The *P2Y purinoceptor 13-like* gene was also downregulated, which has been reported to regulate lung endothelial barrier integrity in humans (137). Multiple MF GO terms associated with DNA binding including binding for transcription and receptor activity in the spleen indicate an active innate inflammatory immune response through modulation of macrophages and hence cytokine activity and histamine release. While increased epithelial proliferation ostensibly at the gill surface is characterized by negative regulation of the *delta-like protein 4* and *protein jagged-2-like* in the Notch signaling pathway in the spleen (138).

Interestingly while the systemic innate immune response shows activation through increased gene expression, the inflammatory response is decreased distal to the AGD lesion at the gill in an inflammatory gradient progressing away from the site of the lesion as indicated by the downregulation of a *5-hydroxytryptamine receptor 7-like*, a serotonin receptor on the membranes of immune cells including dendrites, monocytes, macrophages, microglia and lymphocytes (139). Four extracellular and plasma membrane GO terms were enriched among downregulated genes associated with toxin sequestration, humoral immunity through B-cell homeostasis, and tumor invasion. These genes were *saxiphilin-like*, *TNF receptor superfamily member 13B* and *matrix metalloproteinase-28-like* respectively (140–142). Matrix metalloproteinases are also responsible for extracellular matrix degradation involved in tumor invasion and progression (143). A total of 18 metalloproteinases were identified in the enriched GO term ‘metallopeptidase activity’ which included *stromelysin-3-like* (144). Mucus production and wound healing metabolic processes are also upregulated in the gill, particularly transferases associated with glycoprotein production, a key component of mucus (145). Differential gene expression of mucins in AGD has previously been reported in proximity to gill lesions in AGD in Atlantic salmon (16). Active suppression of parasite invasion is also indicated through upregulation of G-protein coupled receptors including *integrin-β-3* (146), and regulatory genes such as *G-protein signaling 21-like* (147) in the GPCR signaling pathway. Integrins mediate cellular adhesion processes and active immune cells and upregulation of these genes has been reported in AGD-affected Atlantic salmon (148). Collectively, the differential gene expression, KEGG pathway and network analyses show significant gene expression impacts on the host immune response and identified prospective key genes/regulators and pathways that may be modulated by *N. perurans* to promote AGD pathogenesis.

### Parasite Invasion and Contact Dependent Cysteine Proteases

Pathogen tissue invasion may be facilitated through the secretion of proteins including toxins, adhesion molecules, effector proteins and enzymes (149) with pathogenicity inferred by both contact and non-contact mechanisms (150). **Figure 5** suggests a model for host parasite interaction based on the differential expression and network analyses in this study where only contact mechanisms appear to be in effect. This includes proteins to facilitate the destruction of the mucus barrier, apoptosis of epithelial cells and re-arrangement of the host actin cytoskeleton to gain access to host tissue. In protozoan and sporozoite parasites cysteine proteases, such as *cathepsin B* identified in our study are critical for contact dependent host invasion through proteolysis of host extracellular matrix proteins and degradation of host immune proteins, including the amoeba genus *Naegleria* and *E. histolytica* (98, 150, 151)*. Cathepsin B* expression in the parasite *Giardia duodenalis*, is induced by soluble host factors that deter attachment (105). However instead of deterring attachment the interaction results in upregulation of expression of *Giardia duodenalis* virulence factors, ultimately enabling parasite attachment in human gastroenteritis (105). The interaction between the host and *N. perurans cathepsin B* needs to be explored further, as it may provide an opportunity to develop alternative treatments for AGD. For example, in *Trypanosoma and Toxoplasma* sp., cathepsin B has been reported to be essential for survival due to cathepsins role in digesting tissue to provide essential nutrients from the host such as iron (152). Cathepsin B protease inhibition through RNAi, vaccination and chemotherapeutics are widely studied for the prevention of cathepsin B-mediated trematode tissue invasion (fluke) in livestock (153) and may provide a starting point for exploring novel AGD therapeutics. Likewise, tumor invasion in many human cancers is facilitated through over expression of cathepsin (154). A wide variety of cathepsin inhibitors have been developed and investigated in human cancers, however evidence suggests their action may be to potentiate other therapeutics in cancer treatment by facilitating membrane passage to induce apoptosis and necrosis of invading cells (154). Targeted therapeutic strategies that facilitate passage into *N. perurans* such as cathepsin mediated cell entry may assist in reducing the toxicity of alternative novel treatments to the host by reducing dosage.

### Parasite Gene Candidates for Remodeling of Gill Epithelia During Attachment

Histopathology of gill tissue affected by AGD has been reported to show re-modeling of host tissue (5, 7, 155). This is characterized by hyperplasia of the lamellar epithelium, fusion of adjacent lamellae and formation of interlamellar lacunae or vesicles, hyperplasia, and hypertrophy of mucus cells. This results in increased production of gill mucus and proliferation of the lamellar epithelium (5). In the proposed host-parasite interaction model for AGD in Atlantic salmon, we suggest that re-modeling of the gill tissue may be similar to that observed for *E. histolytica* in amoebiasis in humans (hsa05146). In amoebiasis, *E. histolytica* facilitates actin rearrangement of the cytoskeleton through binding with host fibronectin (FN) which stimulates protein kinase C (PKC) or protein kinase A (PKA) pathways for rearrangement of actin in the amoebae cytoskeleton in preparation for attachment (156, 157). *PKC* and several *actin* and regulatory component genes were isolated in *N. perurans* (**Supplementary Tables 12** and **14**), however none were differentially expressed. Similarly, only some of the components necessary for lesion formation (*vinculin*, *α-actinin*, *tropomyosin*, and *myosin I*) in *E. histolytica* are present in our dataset (157).

Prokumamolisin was identified in our *de novo* assembly of unmapped reads through the transcript annotated as *prokumamolisin activation domain containing protein* which is an orthologue from *Acanthamoeba castellanii* (112). It is a sedolisin or serine-carboxyl peptidase, which is a proteolytic enzyme that has been reported to be secreted into extracellular space by the pathogenic amoebae *Acanthamoeba castellanii*, but not by non-pathogenic amoebae (112). Interestingly, prokumamolisin is the inactive form of kumamolisin that acts as a collagenase (158). This suggests prokumamolisin may function in re-modeling of the host epithelia during attachment in *N. perurans*. *Entamoeba histolytica* adhesion, migration and phagocytosis is controlled through engagement with a *p-21 activated kinase* (159) which was also upregulated in our dataset (*NpPAK*). Interestingly the host gene co-expression network may provide the answer to lesion formation through Atlantic salmon expression of a C-reactive protein, hemagglutinin/amebocyte aggregation factor-like (HAAF). In *Limulus polyphemus* HAAF induces aggregation of amoebae and binding through interaction with limunectin and an endotoxin binding protein (90). These then induce blood coagulation, complement and adhesion processes by the host to repair the tissue at the affected site.

### Gene Candidates for Modulation of the Parasites Local Environment

In our study, we identified candidate genes that suggest the amoeba may defend itself against ROS production thereby circumventing this host defense mechanism. On attachment ubiquitination pathways and ROS detoxification are upregulated in *Giardia duodenalis* (105). Similar expression patterns are also observed in our study with downregulation of *cathepsin B* in the lesion and the upregulation of *hybrid cluster protein* (*NpHCP*). This host evasion mechanism has also been observed in *Entamoeba histolytica* during invasion of tissue in amoebiasis in humans (160). In parasites, proteosomes have roles in virulence, toxin production, differentiation, cell cycle, proliferation, and encystation during the invasion of host tissue (104, 161). The ubiquitin proteasome system is represented by the upregulated gene candidate *Fbox domain containing protein*. Other mechanisms by which amoeba may control their environment include altering the local pH by acidification of the gill tissue creating a safe niche for proliferation. This occurs through the release of protons by expression of a *vacuolar H^+^-ATPase* (*NpV-ATPase*) which has been observed in *Leishmania* promastigotes and *Plasmodium falciparum* parasites (102, 103). In AGD, acidification resulting from proton release is likely to benefit *N. perurans* by reducing ammonium toxicity as it is released from the gills (162) and may also assist in mucus degradation.

### Parasite Pathogenicity Gene Candidates

Another form of contact-dependent invasion is observed in *Naegleria* sp., *Acanthamoeba* sp. and *Entamoeba* sp. where cytoplasmic extensions form a phagocytic amoebastome or food cup which enables pathogen actin genes to interact with host heat shock proteins in a pathogenic manner (150, 163). In *N. perurans*, an alternative theory to the amoebastome for interacting with the host heat shock protein (HPSB1) is the surface protein expressed in this study, *prohibitin* (*NpPHB*). This protein has been shown to bind to heat shock protein 70 (Hsp70) in the host-pathogen interaction between *Leishmania donovani* and macrophages in the human disease leishmaniasis (113). Prohibitin has been reported to increase infectivity by increasing protein surface density and binding to Hsp70 (134, 135). In parasites (and other protozoans) a switch in morphology is also reported to increase pathogenicity (164). To date, *N. perurans* has been reported to revert to pseudocyst morphology under stress, otherwise it maintains normal trophozoite morphology in *in vitro* cultured conditions (23). However, conformational changes in morphology may remain a plausible component of virulence for *N. perurans* in AGD but this theory needs to be explored further.

### Gene Candidates for Virulence and Pathogenicity

*AprA* is a metalloprotease gene known to be a virulence factor in *Pseudomonas entomophila* however it is also able to suppress induction of host antimicrobial peptides (111, 165). The closest species blast match for *NpAprA* was *Capsaspora owczarzaki*, a unicellular amoeba protist rather than a bacterial species suggesting this is a candidate for *N. perurans* protection against the host immune response, and not a contaminant from the commensal bacteria. *AprA* expression is regulated by the GacS/GacA two component virulence system of *P. entomophila* and other species (111). This system is responsible for the production of multiple virulence factors including pore-forming toxins. In *N. perurans*, *NpAprA* expression may be regulated by the analogous two component PhoP/Q system (*PhoPQ*) which was detected in our dataset and is a master regulator of virulence in *Salmonella enterica* and *Pectobacterium versatile* (106). In *Salmonella enterica*, *PhoPQ* was acquired through lateral gene transfer (106). Both lateral and horizontal gene transfer have been reported between bacteria and eukaryotes including the amoeba, *Acanthamoeba castellanii*, as well as endosymbionts and their hosts (166–168). In *N. perurans*, the closest species blast match for *NpPhoPQ* was the amoebozoan cellular slime mold *Cavenderia fasciculata* (*syn*. *Dictyostelium fasciculatum*) providing support for the hypothesis that the PhoP/Q system is a candidate for master regulator of virulence in *N. perurans*. Several other gene candidates were identified with potential roles as pathogenic factors including the *MADS-box transcription factor* (*NpMADS*) with similarity to the amoebozoan cellular slime mold *Tieghemostelium lacteum* (*syn. Dictyostelium lacteum*). These TFs are highly conserved in eukaryotes and have a diversity of functional roles including pathogenicity (107, 108). The downregulation of *NpMADS* late in AGD in our study is consistent with this proposed gene candidate roles in AGD when virulent protein secretion may not be required. An endosomal sorting complex gene candidate, *vacuolar sorting protein SNF7* (*NpSNF7*) was identified in the *de novo* lesion with blast match similarity to *Acanthamoeba castellanii* (114). This gene candidate is reported to export O-polysaccharides without secretion tags from the cytosol (114). The final virulence factor candidate is from the Rab family of GTPases, *GTPase activator protein for Ras-like GTPase*, which has been reported to regulate amoebapore virulence factors (160). These form pores in host cells to trigger cell death and degradation of the extracellular matrix. This facilitates host invasion whilst evading the subsequent immune response (160).

### Do Commensal Bacteria Have a Role in AGD Pathogenicity?

The commensal bacteria associated with the lesion add another layer of complexity to the host-pathogen interaction in AGD (33). Bacterial taxa are ubiquitous throughout the marine aquaculture environment, including on the gill surface of salmon where the assemblage present plays a key role in health and physiological functions (169). As both the gill surface and *N. perurans* contain a rich community of microbiota, it is pertinent to examine the bacterial species, which may be implicated in AGD progression at the host-pathogen interface. Bacterial reads in the current study were opportunistically assessed for descriptive purposes, as bacterial mRNA is not polyadenylated at the 3’ end of the transcript, a typically necessary feature for successful reverse transcription for sequencing (47). Some consideration should be made to any possible bias of this sequencing chemistry, including potential for increased incidences of species with lower GC content.

A parallel *in vivo* investigation of the bacterial community present on AGD-affected gill tissue of salmon from the same cohort as the current study determined that a known pathogenic bacterium *Tenacibaculum dicentrarchi* was heavily abundant within the AGD lesioned tissue (33). Other field and lab-based AGD studies have demonstrated the significant presence of specific taxa, including the genus *Psychroserpens* (170), *Winogradskyella* and *Staphylococcus* (171) implicated in AGD development or linked to the presence of AGD. The Genus *Legionella* is a known pathogenic clade in mammalian systems. Most prominently the species *Legionella feeleii* has been shown to harbor intracellularly within amoebae cells (172). It is possible this species may have been present in concert with the AGD causing *N. perurans* trophozoites, but this requires further substantiation. *Pseudomonas stutzeri* is a functional denitrifying taxon present in both aquatic and terrestrial soils and sediments (173), and was a dominant taxon taken from the bacterial read data. The functional purpose of denitrifying taxa suits colonization on the surface of teleost fish gills due to the constant secretion of nitrogenous waste products over the gill epithelium (174). The higher proportion of annotated genes based on TPM for *P. stutzeri* may alternatively reflect microbial dysbiosis at the gill lesion (**Supplementary Table 13**).

A proportion of identified bacterium were of marine origin, meaning that there is relevance in assessing the species sequenced and their function. The majority of bacterial taxa identified in the gill lesion *N. perurans* transcriptome were also in the unmapped gill transcriptome. However, several new taxa were identified after addition of naïve and distal to the lesion biopsy raw reads to the lesion transcriptome assembly. Most notably, *Nocardia jejuensis* only found in AGD-affected fish which is a novel *Nocardia* species from South Korea with 97.4% similarity to *Nocardia salmonicida* (175). Nocardia infections are generally thought to be opportunistic, however other species from the Genus including *Nocardia seriolae*, the causal agent of nocardiosis is known to cause gill nodules together with other clinical disease symptoms in yellowtail and amberjack aquaculture (176). In Atlantic salmon *Nocardia asteroides* has been isolated from skin lesions in tank reared animals (177). The *Flavobacteriales* Order contains multiple marine pathogenic bacteria including *Fleixbacter* and *Tenacibaculum* (178). The relatively even proportion of this bacterium identified across AGD-affected and naïve fish suggests that these species are part of the normal microbiota and not related specifically to the occurrence of AGD in this study. It is important to note that Genus level identifications may be more relevant than species in the unmapped transcriptome due to the low coverage of contigs greater than 500 bp.

The detection of *Halomonas halocynthiae* is also pertinent as an exclusive marine inhabiting species, which has been isolated from the gill surface of other marine organisms (179). This genus has also been identified in concert with *in vitro* cultured *N. perurans* in amplicon sequencing studies (26). Similarly, *Adhaeribacter aquaticus* is another species typically associated with aquatic biofilm environments and is likely present in this dataset as a commensal taxon. Another species commonly associated with aquatic bioreactor communities is *Leptospirillum ferriphilum*, which was identified on the gill (180). This iron-oxidizing species may be a functional commensal taxon utilizing available resources within the cellular respiration and oxygen transfer mechanisms of the gill. The assessment of bacterial reads in the current study has identified several taxa which may play functional roles either on the gill surface, or during AGD pathogenesis. Whether the severity of *N. perurans* as the primary pathogen responsible for AGD is altered by specific commensal bacteria remains to be determined as future studies examine the interaction between the host, the bacteria and *N. perurans* in AGD.

In our study we characterized the molecular events 21 days post-AGD induction in the host and parasite to identify gene candidates to propose a model for host-parasite interaction during pathogenesis of AGD in Atlantic salmon. In *N. perurans* multiple gene candidates are upregulated that are indicators for virulence and regulation of virulence in other species. A comparison of the gene networks between diseases states (AGD v non-AGD) in host tissue indicates that genes expressed in the wnt-pathway are negatively impacted during AGD as observed in disease states in other species where tissue invasion is a factor. Genes candidates associated with evasion of host defense mechanisms and formation of the mucoid lesion were upregulated. This study also presents the first transcriptomic study of the causative agent for AGD, *N. perurans* providing a new resource to contribute to better understanding of the parasite. These gene candidates, networks and pathways may be explored further to develop new hypotheses for future AGD research, and may lead to new therapeutics for AGD in the future.

## Data Availability Statement

The original contributions presented in the study are publicly available. This data can be found here: https://www.ncbi.nlm.nih.gov/geo/query/acc.cgi?acc=GSE166760.

## Ethics Statement

The animal study was reviewed and approved by Commonwealth Scientific and Industrial Research Organisation Queensland Animal Ethics Committee under applications 2018-09, 2017-35, and 2017-36.

## Author Contributions

AM, NB, and JW contributed to conception and design of the study. JS organized and collected the samples. NB, PL, and AM performed the data acquisition. AM performed the host bioinformatic/systems biology analyses. NB performed all pathway visualization/interpretation and the parasite bioinformatic analyses. NB interpreted the data and wrote the first draft of the manuscript. NB, JW, JS, and AM wrote sections of the manuscript. All authors contributed to manuscript revision, read, and approved the submitted version.

## Funding

This work was funded by CSIRO Agriculture and Food Science Investment Project 252 (OD-206222).

## Conflict of Interest

The authors declare that the research was conducted in the absence of any commercial or financial relationships that could be construed as a potential conflict of interest.

## Supplementary Material

Supplementary Table 1 Illumina sequencing statistics. Data yield, number of obtained raw paired-end reads, number of mapped fragments for each RNA-Seq library [Excel table].

Supplementary Table 2 Top differentially expressed genes (-log_10_FDR > 5) in the gill. **(A)** Gill lesion (L) of an AGD-affected fish compared to a gill biopsy in a naïve (C) Atlantic salmon. **(B)** Gill lesion biopsy compared to a biopsy distal (D) to the lesion of an AGDaffected fish. **(C)** Gill biopsy distal to the lesion of an AGD-affected fish compared to a gill biopsy in a naïve Atlantic salmon. [Excel table]

Supplementary Table 3 Enriched gene ontology (GO) terms for the local response to AGD at the gills of Atlantic salmon at the lesion, distal to the lesion and in naïve fish. [Excel table]

Supplementary Table 4 Top differentially expressed genes (-log_10_FDR > 5) in the head kidney of an AGD-affected fish (A) compared to a naïve (C) Atlantic salmon. [Excel table].

Supplementary Table 5 Top differentially expressed genes (-log_10_FDR > 5) in the spleen of an AGD-affected fish (A) compared to a naïve (C) Atlantic salmon. [Excel table].

Supplementary Table 6 Enriched gene ontology (fold change > 2; P < 0.05) for the systemic response to amoebic gill disease in the head kidney of Atlantic salmon affected by AGD in contrast to naïve fish. [Excel table].

Supplementary Table 7 Enriched gene ontology (fold change > 2; P < 0.05) for the systemic response to amoebic gill disease in the spleen of Atlantic salmon affected by AGD in contrast to naïve fish. [Excel table].

Supplementary Table 8 Key transcription factors (TFs) and their regulatory impact factor (RIF) score in amoebic gill disease in Atlantic salmon. [Excel table].

Supplementary Table 9 Differential expression of host transcription factors (TFs) (fold change > 2; P < 0.05) in response to amoebic gill disease pathology in Atlantic salmon. [Excel table].

Supplementary Table 10 Differential expression of genes and transcription factors (TFs) (fold change > 2; P < 0.05) in the *zinc finger OZF-like* gene network. [Excel table].

Supplementary Table 11 Differential expression of *Neoparamoeba perurans* genes (fold change < 2; P < 0.05) in the lesion of an amoebic gill disease affected fish in comparison to cultured trophozoites. [Excel table].

Supplementary Table 12
*Neoparamoeba perurans* genes identified in the *de novo* assembly of the unmapped Atlantic salmon amoebic gill disease lesion data. [Excel table].

Supplementary Table 13 Bacterial species and their genes identified in the unmapped Atlantic salmon amoebic gill disease data. [Excel table].

Supplementary Table 14 Genes identified in the host-parasite response to amoebic gill disease in Atlantic salmon (host) and *Neoparamoeba perurans* (pathogen). The model is shown in Figure 4. [Excel table].

Supplementary Figure S1 Differential gene expression in the gills in response to amoebic gill disease. **(A)** Level of agreement among the biological replicates of a naïve (n=4; red, CG), AGD-affected tissue distal to the AGD lesion (n=4; green, DG), and a lesion on a gill affected by AGD (n=3; blue, LG). The heat map shows the hierarchically clustered Spearmancorrelations resulting from comparing normalized expression for all gill samples against each other. Sample clustering indicates the consistency between the biological replicates. **(B–D)** Volcano plots of differentially expressed genes in the gill, highlighting genes with a minimum 2-fold change in expression and false discovery rate greater than five comparing AGD lesion to a naïve fish **(B)**; the lesion compared to the region distal to the lesion of an AGD-affected fish **(C)**; and the region distal to the lesion of an AGD-affected fish compared to a naïve fish **(D)**. [word doc]

Supplementary Figure S2 Differential gene expression in the head kidney and spleen in response to amoebic gill disease. Level of agreement among the head kidney (HK) **(A)** and spleen (SP) (B) biological replicates of a naïve (n=4; C; red) and an AGD-affected Atlantic salmon (n=4; A; blue). The heat map shows the hierarchically clustered Spearman correlations resulting from comparing normalized expression for HK or SP samples against each other. Sample clustering indicates consistency between biological replicates. Volcano plots of differentially expressed genes in the HK **(C)** and SP **(D)**, highlighting genes with a minimum 2-fold change in expression and false discovery rate greater than five comparing AGD lesion to a naïve fish. [word doc]

Supplementary Figure S3 Enriched gene ontology (GO) terms (hypergeometric test, Bonferroni-adjusted P < 0.05) among the upregulated **(A)** and downregulated **(B)** differentially expressed genes in the biopsy distal to the lesion. The majority of the upregulated enriched terms are related to host and immune response and downregulated to organism developmental processes. [word doc]

Supplementary Figure S4 Differential gene expression in *Neoparamoeba perurans* in the lesion of amoebic gill disease in Atlantic salmon compared to cultured *N. perurans* trophozoites. **(A)** Heat map shows hierarchical clustering of differentially expressed genes (rows) with differential expression among replicates from a gill biopsy of the lesion (LES) and cultured trophozoites (CUL) from *N. perurans*. Expression values are log2-transformed and median-centered by gene. **(B)** Volcano plot of differentially expressed genes in the gill biopsy lesion, highlighting genes with a minimum 2-fold change in expression (P < 0.05) compared to in vitro cultured *N. perurans* trophozoites. [word doc]
