# Supplementary material for: Host-Parasite Interaction of Atlantic salmon (Salmo salar) and the Ectoparasite Neoparamoeba perurans in Amoebic Gill Disease
Source: Front Immunol. 2021 May 31;12:672700. doi: 10.3389/fimmu.2021.672700 (PMC8202022; doi:10.3389/fimmu.2021.672700)
Supplement: Supplementary Figure S1 — Differential gene expression in the gills in response to amoebic gill disease. (A) Level of agreement among the biological replicates of a naïve (n=4; red, CG), AGD-affected tissue distal to the AGD lesion (n=4; green, DG), and a lesion on a gill affected by AGD (n=3; blue, LG). The heat map shows the hierarchically clustered Spearmancorrelations resulting from comparing normalized expression for all gill samples against each other. Sample clustering indicates the consistency between the biological replicates. (B–D) Volcano plots of differentially expressed genes in the gill, highlighting genes with a minimum 2-fold change in expression and false discovery rate greater than five comparing AGD lesion to a naïve fish (B); the lesion compared to the region distal to the lesion of an AGD-affected fish (C); and the region distal to the lesion of an AGD-affected fish compared to a naïve fish (D). [word doc] [file Image_1.pdf]

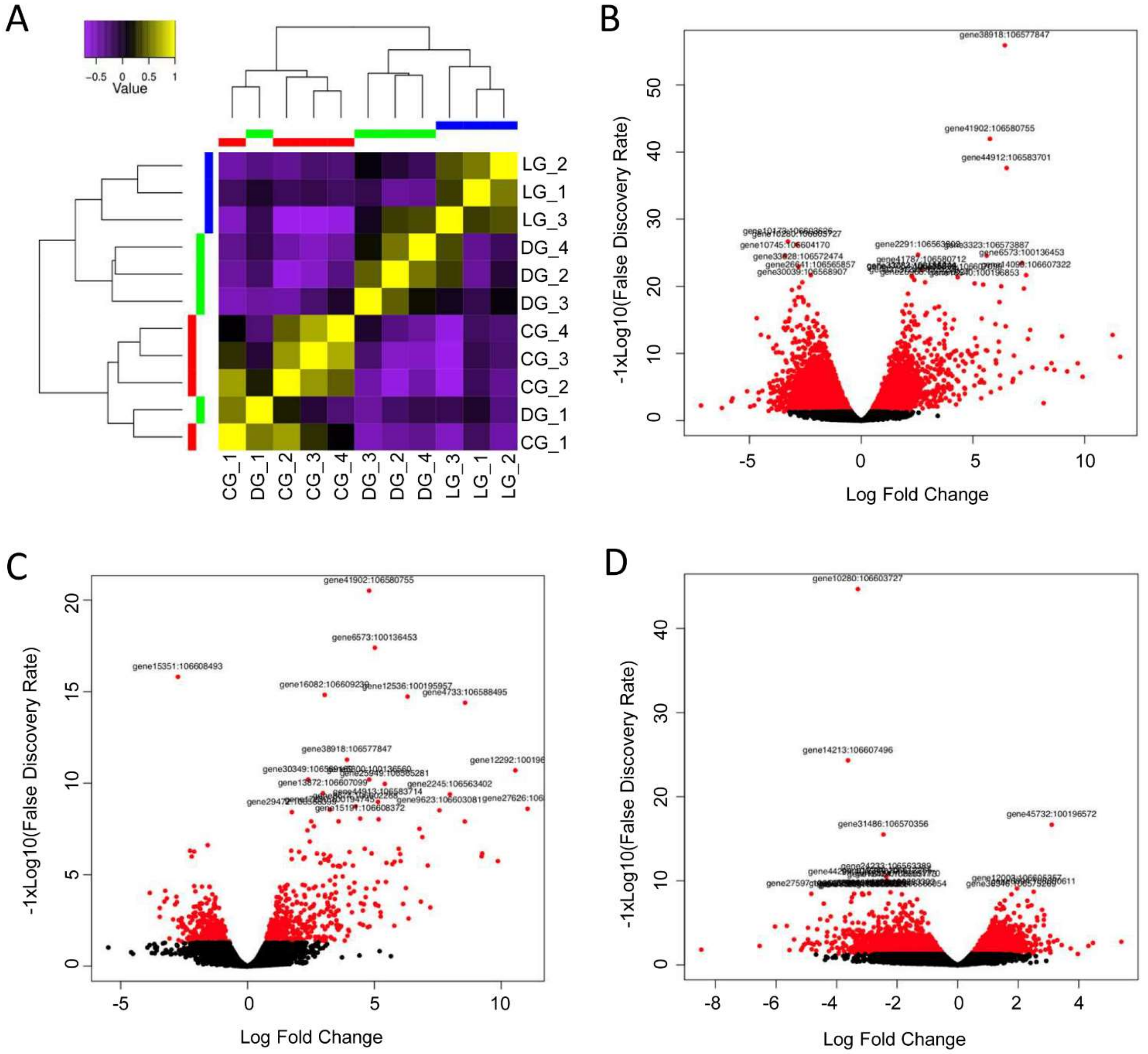

Supplementary Figure 1. Differential gene expression in the gills in response to amoebic gill disease. **(A)** Level of agreement among the biological replicates of a naïve (n=4; red, CG), AGD-affected tissue distal to the AGD lesion (n=4; green, DG), and a lesion on a gill affected by AGD (n=3; blue, LG). The heat map shows the hierarchically clustered Spearman correlations resulting from comparing normalized expression for all gill samples against each other. Sample clustering indicates the consistency between the biological replicates. **(B-D)** Volcano plots of differentially expressed genes in the gill, highlighting genes with a minimum 2-fold change in expression and false discovery rate greater than five comparing AGD lesion to a naïve fish **(B)**; the lesion compared to the region distal to the lesion of an AGD-affected fish **(C)**; and the region distal to the lesion of an AGD-affected fish compared to a naïve fish **(D)**.
