# Supplementary material for: Host-Parasite Interaction of Atlantic salmon (Salmo salar) and the Ectoparasite Neoparamoeba perurans in Amoebic Gill Disease
Source: Front Immunol. 2021 May 31;12:672700. doi: 10.3389/fimmu.2021.672700 (PMC8202022; doi:10.3389/fimmu.2021.672700)
Supplement: Supplementary Figure S2 — Differential gene expression in the head kidney and spleen in response to amoebic gill disease. Level of agreement among the head kidney (HK) (A) and spleen (SP) (B) biological replicates of a naïve (n=4; C; red) and an AGD-affected Atlantic salmon (n=4; A; blue). The heat map shows the hierarchically clustered Spearman correlations resulting from comparing normalized expression for HK or SP samples against each other. Sample clustering indicates consistency between biological replicates. Volcano plots of differentially expressed genes in the HK (C) and SP (D), highlighting genes with a minimum 2-fold change in expression and false discovery rate greater than five comparing AGD lesion to a naïve fish. [word doc] [file Image_2.pdf]

frontiers

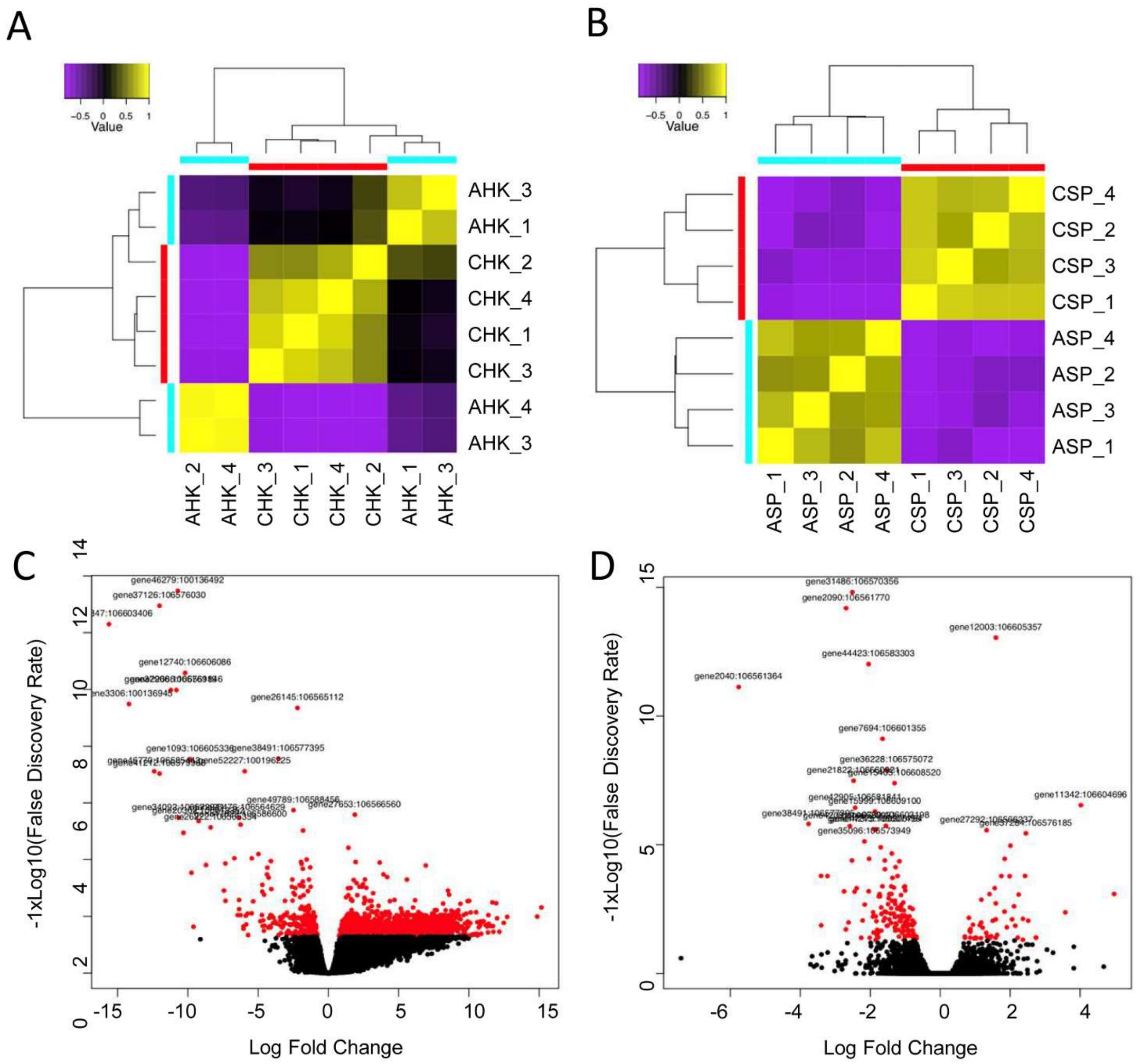

Supplementary Figure 2. Differential gene expression in the head kidney and spleen in response to amoebic gill disease. Level of agreement among the head kidney (HK) **(A)** and spleen (SP) **(B)** biological replicates of a naïve (n=4; C; red) and an AGD-affected Atlantic salmon (n=4; A; blue). The heat map shows the hierarchically clustered Spearman correlations resulting from comparing normalized expression for HK or SP samples against each other. Sample clustering indicates consistency between biological replicates. Volcano plots of differentially expressed genes in the HK **(C)** and SP **(D)**, highlighting genes with a minimum 2-fold change in expression and false discovery rate greater than five comparing AGD lesion to a naïve fish.
