# Supplementary material for: Host-Parasite Interaction of Atlantic salmon (Salmo salar) and the Ectoparasite Neoparamoeba perurans in Amoebic Gill Disease
Source: Front Immunol. 2021 May 31;12:672700. doi: 10.3389/fimmu.2021.672700 (PMC8202022; doi:10.3389/fimmu.2021.672700)
Supplement: Supplementary Figure S3 — Enriched gene ontology (GO) terms (hypergeometric test, Bonferroni-adjusted P < 0.05) among the upregulated (A) and downregulated (B) differentially expressed genes in the biopsy distal to the lesion. The majority of the upregulated enriched terms are related to host and immune response and downregulated to organism developmental processes. [word doc] [file Image_3.pdf]

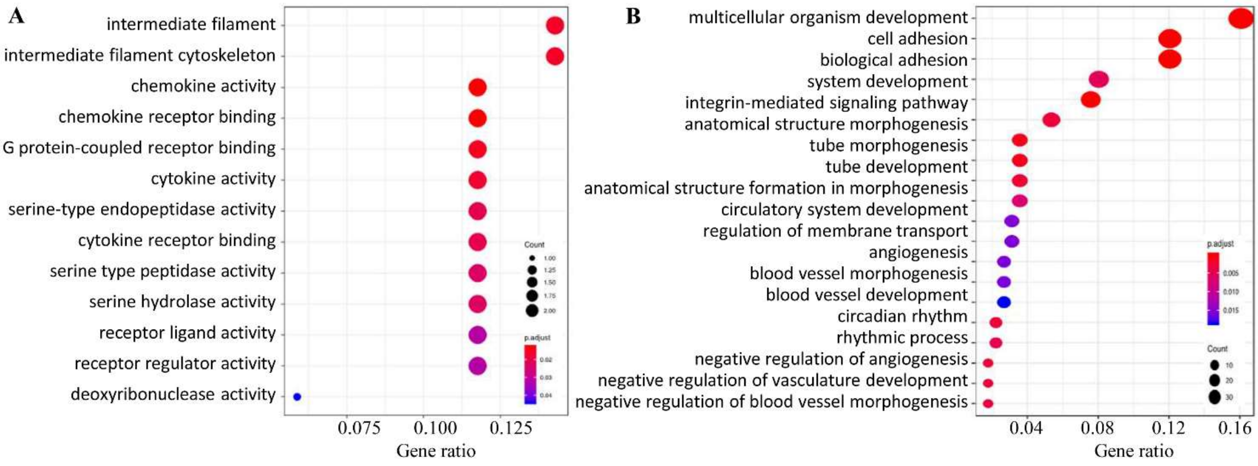

Supplementary Figure 3. Enriched gene ontology (GO) terms (hypergeometric test, Bonferroni-adjusted $P < 0.05$) among the upregulated **(A)** and downregulated **(B)** differentially expressed genes in the biopsy distal to the lesion. The majority of the upregulated enriched terms are related to host and immune response and downregulated to organism developmental processes.
