# Supplementary material for: Host-Parasite Interaction of Atlantic salmon (Salmo salar) and the Ectoparasite Neoparamoeba perurans in Amoebic Gill Disease
Source: Front Immunol. 2021 May 31;12:672700. doi: 10.3389/fimmu.2021.672700 (PMC8202022; doi:10.3389/fimmu.2021.672700)
Supplement: Supplementary Figure S4 — Differential gene expression in Neoparamoeba perurans in the lesion of amoebic gill disease in Atlantic salmon compared to cultured N. perurans trophozoites. (A) Heat map shows hierarchical clustering of differentially expressed genes (rows) with differential expression among replicates from a gill biopsy of the lesion (LES) and cultured trophozoites (CUL) from N. perurans. Expression values are log2-transformed and median-centered by gene. (B) Volcano plot of differentially expressed genes in the gill biopsy lesion, highlighting genes with a minimum 2-fold change in expression (P < 0.05) compared to in vitro cultured N. perurans trophozoites. [word doc] [file Image_4.pdf]

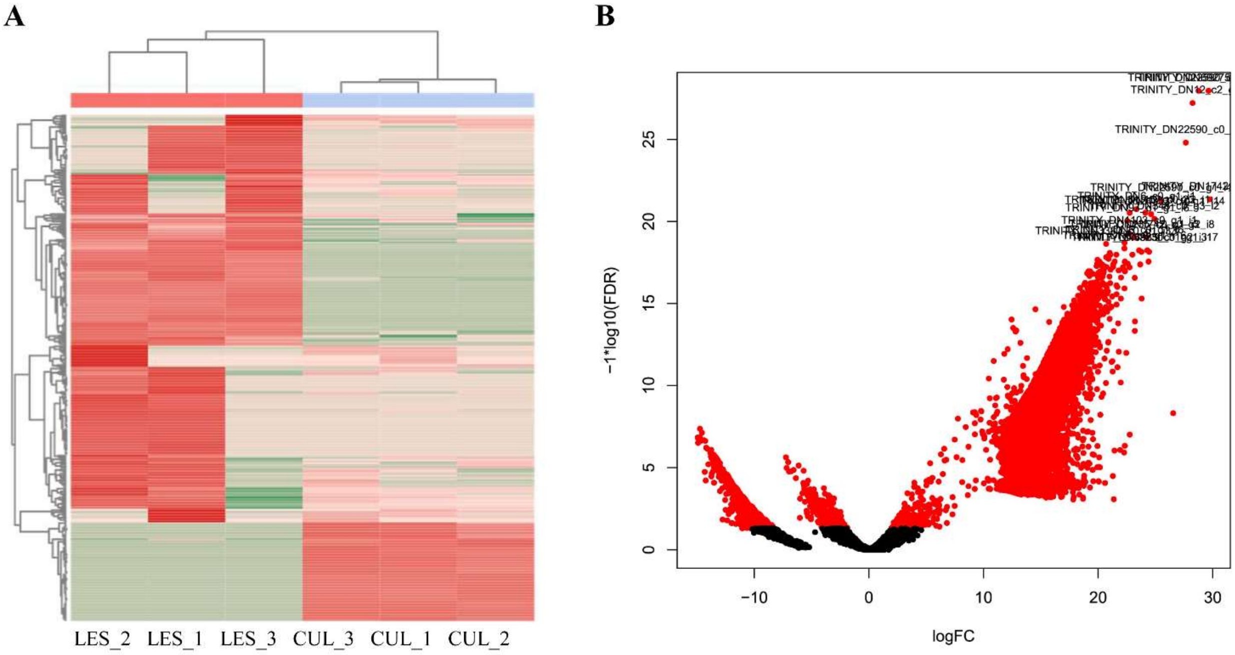

Supplementary Figure 4. Differential gene expression in *Neoparamoeba perurans* in the lesion of amoebic gill disease in Atlantic salmon compared to cultured *N. perurans* trophozoites. **(A)** Heat map shows hierarchical clustering of differentially expressed genes (rows) with differential expression among replicates from a gill biopsy of the lesion (LES) and cultured trophozoites (CUL) from *N. perurans*. Expression values are log2-transformed and median-centered by gene. **(B)** Volcano plot of differentially expressed genes in the gill biopsy lesion, highlighting genes with a minimum 2-fold change in expression ($P < 0.05$) compared to in vitro cultured *N. perurans* trophozoites.
